# Deciphering the Role of ASPM in Breast Cancer: A Comprehensive Multicohort Study

**DOI:** 10.3390/cancers16223814

**Published:** 2024-11-13

**Authors:** Asmaa Ibrahim, Nehal M. Atallah, Shorouk Makhlouf, Michael S. Toss, Andrew Green, Emad Rakha

**Affiliations:** 1 Academic Unit for Translational Medical Sciences, School of Medicine, University of Nottingham Biodiscovery Institute, University Park, Nottingham NG7 2RD, UK; mzxai2@nottingham.ac.uk (A.I.); mzxna4@exmail.nottingham.ac.uk (N.M.A.); mzxsm9@exmail.nottingham.ac.uk (S.M.); mrzarg@exmail.nottingham.ac.uk (A.G.); 2 Department of Pathology, Faculty of Medicine, Suez Canal University, Ismailia P.O. Box 41522, Egypt; 3 Department of Pathology, Faculty of Medicine, Menoufia University, Shebeen El-Kom P.O. Box 32951, Egypt; 4 Department of Pathology, Faculty of Medicine, Assiut University, Assiut P.O. Box 7111, Egypt; 5 Histopathology Department, Royal Hallamshire Hospital, Sheffield Teaching Hospitals NHS Foundation Trust, Sheffield S10 2TN, UK; msamt9@exmail.nottingham.ac.uk; 6 Pathology Department, Hamad Medical Corporation, Doha P.O. Box 3050, Qatar

**Keywords:** ASPM, invasive breast cancer, prognosis

## Abstract

Background: Assembly factor for spindle microtubules (ASPM) has gained significant attention in cancer research due to its association with tumor growth and progression. Through the analysis of large-scale genomic datasets, ASPM has been identified as the top upregulated gene in breast cancer (BC), characterized by high proliferation. This multicohort study aimed to investigate the clinicopathological and prognostic significance of ASPM mRNA and protein expression in BC. Methods: ASPM mRNA expression was assessed using the Cancer Genome Atlas (TCGA) BC cohort and has been further validated in the Molecular Taxonomy of BC International Consortium (METABRIC) (*n* = 1980), The Uppsala cohort (*n* = 249), in addition to the combined multicentric cohort (*n* = 7252). ASPM protein expression was evaluated in a large BC cohort (*n* = 1300) using immunohistochemistry. The correlations between ASPM expression, clinicopathological parameters, molecular subtypes and outcome were assessed. The response to taxane treatment was compared to the clinical prognosis of ASPM using the ROC plotter. Results: High ASPM mRNA and protein expression were significantly associated with aggressive BC features and poor survival across all cohorts. The association with poor outcomes was maintained in the adjuvant chemotherapy and radio-therapy-treated patients. Responders to taxane treatment showed significantly elevated ASPM levels compared to non-responders. Conclusions: High ASPM expression predicts poor prognosis in BC. It may play a role in treatment resistance within a specific subgroup of patients. Further clinical trials are warranted to explore the potential of ASPM as a target for therapeutic interventions in cancer.

## Simple Summary

ASPM is a gene involved in cell division, and recent research shows that it plays an important role in breast cancer (BC). This study looked at large datasets of BC patients to see how ASPM is linked to tumor growth and patient outcomes. We found that patients with higher levels of ASPM had more aggressive forms of cancer and poorer chances of survival. This suggests that ASPM may be useful as a marker to predict how aggressive the BC is and how well it will respond to treatment. These findings could guide future research and potentially lead to new treatments targeting ASPM in cancer.

## 1. Introduction

Despite the significant progress in surgical interventions and comprehensive therapies, a substantial proportion of newly diagnosed breast cancer (BC) patients still face the risk of death and recurrence [1]. Cancer is characterized by abnormal, uncontrolled cell division, making antimitotic therapies a logical choice for treating the abnormal proliferation of transformed cells. These drugs are highly selective and target the mitotic phase of the cell cycle.

Induced mitotic cell death typically involves the inhibition of mitotic progression by disrupting the spindle and restricting the availability and functionality of key mitotic regulatory proteins [2].

Recent research has identified multiple molecules and pathways linked to mitotic cell division [3,4,5], suggesting new potential directions for future treatments for BC.

However, despite the discovery and development of mitosis-selective inhibitors, the incomplete comprehension of these pathways poses a significant challenge to achieving clinical success [6]. Even recently developed antimitotic targeting drugs, such as mitotic kinases and spindle motor proteins, have not been able to demonstrate comparable efficacy in human clinical trials [7].

Bridging the gap between promising preclinical trials and effective translational bedside treatment requires further investigation into the mechanistic pathways of mitotic cell divisions and comprehensive targeted validations on the tissue level [8].

The proper segregation of chromosomes during mitosis is crucial for the accurate transmission of genetic material, and dysregulation of mitosis-regulating complexes and spindle-associated proteins has been linked to malignant progression [9,10]. This would further open possible directions for novel targeted therapies.

The assembly factor for spindle microtubules (*ASPM*) located on chromosome 1q31 has been identified as a key driver gene [11] involved in mitotic spindle regulation, proliferation, neurogenesis and brain size regulation [12,13]. In addition, it acts as a positive regulator of the Wnt signaling pathway, one of the crucial cascades regulation cell proliferation and differentiation [14].

Previous studies have shown that *ASPM* is upregulated in various malignant tumors including hepatocellular carcinoma (HCC) [15,16], non-small cell lung cancer [17], glioblastoma and other high-grade gliomas [12,18,19]. Its overexpression has been linked to poor clinical outcomes and recurrence [12,15,16,17,18,19,20,21]. However, the prognostic and predictive role of *ASPM* in BC remains unknown.

Therefore, the present study aimed to investigate the clinicopathological and prognostic significance of ASPM mRNA and protein expression in BC using large and well-characterized cohorts of BC. This study used a dual approach by integrating large-scale meta-analytic analysis of public datasets with experimental validation.

## 2. Materials and Methods

### 2.1. Discovery Cohort

The whole slide images (WSIs) and data from The Cancer Genome Atlas (TCGA) for BC (BRCA) were utilized. The relevant data can be accessed at https://portal.gdc.cancer.gov/projects/TCGA-BRCA (accessed on 14 October 2020) and https://genome-cancer.ucsc.edu/ (accessed on 14 October 2020).

The inclusion criteria for the study included histologically confirmed BC cases from the TCGA dataset with complete clinical and survival data where patients were aged 18 years or older and samples had sufficient tumor content for analysis. Conversely, patients who underwent neoadjuvant treatment prior to surgery, had incomplete clinical data or follow-up information, or were classified as benign or non-malignant were excluded.

A bioinformatic analysis was used for the selection of key genes associated with high mitotic scores in BC as the most appropriate representative of proliferative activity [11].

WSIs of hematoxylin and eosin (H&E) stained samples from 1053 BC cases were obtained from the TCGA dataset [22]. Mitotic figures were quantified by a primary pathologist as outlined in previous methodologies [23] while a second pathologist assessed 20% of the samples, yielding a strong agreement level (intraclass correlation coefficient, ICC = 0.68, *p* < 0.001). The mitotic count per case was then categorized into high and low groups to predict BC-specific survival (BCSS), as determined using X-TILE software, version 3.6.1 (Yale University School of Medicine, New Haven, CT, USA).

Transcriptomic data from the TCGA were analyzed to identify differentially expressed genes (DEGs) between cases with high and low mitotic scores. Initial data pre-processing was performed, and clinicopathological information such as patient age, histological subtype, and clinical outcome was gathered. The RNA sequencing was performed by the Illumina HiSeq platform (Illumina, Inc., San Diego, CA, USA) and was processed following established protocols [24]. The sequencing reads were aligned to the human hg19 genome using the MapSlice assembly tool (BioNano Genomics, San Diego, CA, USA) [25], and gene expression levels were quantified with RSEM4 for the TCGA GAF 2.13 transcript models with within-sample normalization applied [26]. Genomic copy number (CN) data was inferred using the Affymetrix SNP 6.0 Array (Santa Clara, CA, USA).

### 2.2. Validation Cohorts

#### 2.2.1. The METABRIC Cohort

The dataset from the Molecular Taxonomy of Breast Cancer International Consortium (METABRIC), which includes a total of 1980 BC cases [27], was used to assess mRNA expression and gene CN aberrations. The study had a median follow-up period of 109 months (IQR 62-155). DNA and RNA were isolated from fresh frozen tumor samples, and genomic profiling and transcriptional profiling were performed using the Affymetrix SNP 6.0 and Illumina HT-12v3 platforms, respectively. The gene expression data were pre-processed and normalized as per previous protocols [27].

The study investigated the relationship between mRNA expression and clinicopathological parameters, molecular subtypes, and patient outcomes.

#### 2.2.2. The Uppsala Cohort

The Uppsala cohort included 315 early-stage BC patients, accounting for 65% of all BC surgeries performed in Uppsala County, Sweden, from 1987 to 1989. The cohort had a median follow-up of 126 months (IQR 119-134) [28], and mRNA expression was assessed using Affymetrix U133A and B gene chip microarray profiling, with survival data available for 249 patients. The dataset is accessible through the National Center for Biotechnology Information (NCBI) Gene Expression Omnibus (GEO) under series accession number GSE4922 [29].

#### 2.2.3. Combined Multicentric Cohorts

A multicenter combined cohort of 7252 early-stage BC patients was established by integrating data from 56 publicly available international datasets. This analysis utilized the online tool bc-GenExMiner version 4.0.14 [30]. Supplementary Table S1 includes a comprehensive list of all datasets used to develop the multicenter cohort.

The combined multicenter cohort utilized various microarray platforms, as detailed in Supplementary Table S2. To address the technical variations arising from these different platforms and other factors that may impact data comparability, the gene expression data underwent preprocessing, normalization, and transformation to a common scale, achieving a median of 0 and a standard deviation of 1. These preprocessing steps facilitated the integration of data from all studies included, resulting in the formation of a pooled cohort (see Supplementary Table S3).

### 2.3. Proteomic Study

The Nottingham BC cohort was utilized to evaluate protein expression through immunohistochemistry (IHC). This cohort comprises a large group of primary BC patients (*n* = 1300) who were diagnosed and treated at Nottingham City Hospital in the United Kingdom between 1998 and 2006. Comprehensive clinical data and tumor characteristics were accessible, including the patients’ age at diagnosis, histological tumor type, grade, tumor size, lymph node status, Nottingham Prognostic Index (NPI), and lymph vascular invasion (LVI) [31]. Outcome data were computed, including BC-specific survival (BCSS), which is defined as the duration (in months) from the date of primary surgical treatment to the date of death resulting from BC. Distant metastasis-free survival (DMFS) is defined as the time (in months) from the surgical procedure until the first occurrence of distant metastasis. Information regarding the estrogen receptor (ER), progesterone receptor (PR), and human epidermal growth factor receptor 2 (HER2) was obtained as reported in previous publications [32,33,34,35].The hormonal and HER2 status of the BC samples were assessed in accordance with the guidelines established by the American Society of Clinical Oncology and the College of American Pathologists (ASCO/CAP) [36,37,38]. Cases were categorized according to the molecular classification of BC: (i) Luminal A (ER and/or PR positive, HER2 negative, and Ki67 < 14%); (ii) Luminal B/HER2 negative (ER and/or PR positive, HER2 negative, and Ki67 ≥ 14%); (iii) Luminal B/HER2+ (ER and/or PR positive, HER2 positive); (iv) HER2-enriched (non-luminal) (ER and PR negative, and HER2 positive); and (v) Triple Negative BC (TNBC) (ER, PR, and HER2 negative).

Treatment decisions were made based on tumor characteristics, the Nottingham Prognostic Index (NPI), and hormone receptor status following local protocols. Endocrine therapy was offered to post-menopausal women with ER-positive (ER+) tumors that had moderate or poor NPI scores (>3.4). Patients with ER-negative tumors who were deemed fit for chemotherapy were treated with the classical regimen of cyclophosphamide, methotrexate, and 5-fluorouracil (CMF). Most early-stage BC patients who underwent breast-conserving surgery also received radiotherapy as part of their treatment plan.

### 2.4. Selection of ASPM

DEGs associated with high mitotic scores were determined using the DESeq2 tool in R software (version 3.4.3) with a log2 fold change (≥1 or ≤−1) and a false discovery rate (FDR) < 0.05. Gene Ontology (GO) analysis using the database for annotation, visualization, and integrated discovery (DAVID) online tool (version DAVID 6.8; http://david.ncifcrf.gov/ accessed on 20 March 2024) was conducted with *p*-values < 0.05. The protein–protein interaction (PPI) network was constructed using the STRING website https://string-db.org/ accessed on 20 March 2024 [39] with a minimum required interaction score of high confidence (0.7). CytoHubba, a plugin in Cytoscape software (version 3.7.2), was utilized to rank genes within the network based on network features; employing the density of maximum neighborhood component (DMNC) algorithm to identify the top 10 genes, it calculated the density of the connections around a node. Nodes with high DMNC values are considered central or influential within the network.

### 2.5. Aberrations of Gene Copy Number

Data concerning CN aberrations at the ASPM gene on chr1q31.3 from the Genome-Wide Human SNP Array 6.0 platform (Affymetrix, Santa Clara, CA, USA) profiling (METABRIC cohort) were retrieved. Additionally, hybrid genotyping array platforms (Affymetrix SNP 6.0) used at the Broad Institute to generate TCGA data were utilized for genotyping and CN inference. The correlation between CN aberrations and mRNA expression was evaluated.

### 2.6. Western Blotting

Primary antibody specificity for the ASPM antibody (09-66; Sigma Aldrich, Burlington, MA, USA) was validated by Western blotting using cell lysates of MCF7, MDA-MB-231 and SKBR3 human cell lines obtained from the American Type Culture Collection, Rockville, MD, USA. The ASPM antibody was applied at a dilution of 1:1000 and incubated with the cell lysates. For detection, a 1:1000 dilution of HRP-conjugated secondary antibodies (Dako) was used which showed a specific band. To block non-specific binding, a solution of 5% milk in PBS-Tween (0.1%) (Marvel Original Dried Skimmed Milk, Premier Food Groups Ltd., St. Albans, Hertfordshire, UK) was utilized. The bands corresponding to ASPM were detected using enhanced chemiluminescence (ECL).

### 2.7. Tissue Microarrays and Immunohistochemistry

Representative invasive BC samples previously arranged as tissue microarrays (TMA) using the Grand Master^®^ (3D HISTECH^®^, Budapest, Hungary) were used [40]. To gain insights into the heterogeneity and expression patterns of ASPM in BC, a set of whole tissue sections from 10 cases representing different molecular subtypes containing invasive tumors was used. We aimed to evaluate the distribution and expression of ASPM in various tissue components including malignant tissue, adjacent stroma, and normal terminal duct lobular units (TDLUs).

ASPM protein expression was assessed by immunohistochemistry (IHC) using the Novocastra Novolink polymer detection system (Code: RE7280-K, Leica, Newcastle, UK). Tissue microarrays and full-face sections (4 µm thick) were stained with a rabbit polyclonal antibody against ASPM (09-66; Sigma Aldrich) at a 1:250 dilution for 60 min at room temperature. Antigen retrieval was performed in the citrate buffer (pH 6.0) using a microwave (Whirlpool JT359 Jet Chef 1000 W, Whirlpool Corporation, Benton Harbor, MI, USA.) for 20 min. The chromogenic reaction was developed using 3,3-diaminobenzidine (DAB), and sections were counterstained with hematoxylin. Positive control used human tonsil tissue while negative control was conducted by omitting the primary antibody.

### 2.8. Assessment of ASPM Expression

Tissue microarray (TMA) sections were scanned using the NanoZoomer scanner (Hamamatsu Photonics, Welwyn Garden City, Hertfordshire, UK) at a magnification of 20×, producing high-resolution digital images. The cores with less than 15% tumor epithelial cell content were excluded from analysis. Scoring was performed using the semiquantitative Histo score (H-score) [41]. All cases were scored blind to clinicopathological and outcome data. The cut-offs for dichotomizing protein expression were determined using X-tile bioinformatics software (Yale University, version 3.6.1) [42]. A high cytoplasmic expression of ASPM was defined as an H-score greater than 20 with the corresponding *p*-value and relative risk associated with BCSS adjusted accordingly.

A subset of TMA cores (20%) was randomly chosen and double-scored by a second observer (NA) while 20% of the cases were double-scored by the primary observer (AI) after a washout period to test the inter- and intra-observer reproducibility of the scoring.

To determine whether mRNA expression levels correlated with protein levels and to understand the molecular biology of ASPM protein expression as an end product, we compared ASPM mRNA levels and protein expression in a subset of the Nottingham cases (*n* = 60) that were included in the METABRIC data set [27]. The analysis was performed utilizing cases with both mRNA and protein expression.

### 2.9. Ki67 Staining and Scoring

The IHC expression of Ki67 was assessed as previously described [35]. The IHC staining was performed manually using the Novolink™ Max Polymer Detection Kit (Code: RE7280-K, Leica, Newcastle, UK), according to the manufacturer’s instructions. A specific primary antibody, MIB-1 monoclonal mouse antibody (diluted 1:100), was used and incubated for 1 h at room temperature. The assessment focused on invasive tumor cells, and the scoring was performed as a percentage of positively stained nuclei [43]. A high Ki-67 score was defined when more than 14% of tumor cell nuclei displayed staining, regardless of staining intensity [44].

### 2.10. Statistical Analysis

Statistical analyses were conducted using SPSS v26 (Chicago, IL, USA) for Windows. Student’s *t*-test and analysis of variance (ANOVA) were employed to assess the correlation between ASPM mRNA levels as a continuous variable and various clinicopathological parameters in the METABRIC and TCGA datasets. The cut-off points for dichotomizing mRNA and protein expression into high and low groups were determined using the X-tile bioinformatics software version 3.6.1 (Yale University, New Haven, CT, USA) [42]. *ASPM* threshold value was not uniform across all datasets. The association between ASPM expression and clinicopathological parameters in invasive BC was assessed using the chi-squared test without correction for categorized data, focusing on BCSS.

The degree of inter- and intra-observer agreement was assessed using the intraclass correlation coefficient (ICC) for continuous data. Pearson correlations were performed between ASPM mRNA expression log intensity values and their corresponding protein expression using the H-score. The Mann–Whitney–Wilcoxon test was used to assess the significance of the correlation between copy number and aberrant mRNA expression. Survival rates were determined using the Kaplan–Meier method and compared by the log-rank test. Hazard ratios (HR) and corresponding 95% confidence intervals (CI) were calculated.

Multivariate analysis using the Cox regression model determined the influence of ASPM expression when adjusted to other variables. All statistical tests were performed using a two-tailed approach with a *p*-value of less than 0.05 being statistically significant. This study complied with the reporting recommendations for tumor marker prognostic studies (REMARK) criteria, as detailed in Supplementary Table S4 [45].

## 3. Results

### 3.1. Differential Gene Expression Analysis and the Selection of ASPM

A total of 1776 upregulated genes were identified. Among the upregulated genes, 35 genes were enriched during cell division using Gene Ontology (GO) analysis of the DAVID online tool (Supplementary Table S5). DEGs were then used to predict the PPI network using the STRING website. The PPI network was constructed using 35 upregulated DEGs. These were visualized by Cytoscape (v3.7.2.). Ten were recognized as hub genes, with ASPM ranking at the top of the list. These hub genes include (ASPM, PTTG1, NCAPG, KIFC1, SGO1, CEP55, CDCA5, KIF2C, CDCA8, and CDCA2), and the potential gene interactions are presented in Supplementary Figure S1.

### 3.2. ASPM Gene Copy Number Aberrations and mRNA Levels

In the TCGA cohort, 768/1063 (72%) had CN gain for ASPM, while 16/1063 (1.5%) showed CN loss. In the METABRIC dataset, CN gain was observed in 867/1980 (43.8%) of cases, while CN loss was observed in only 2/1980 (0.1%) of cases.

**Figure 1:**
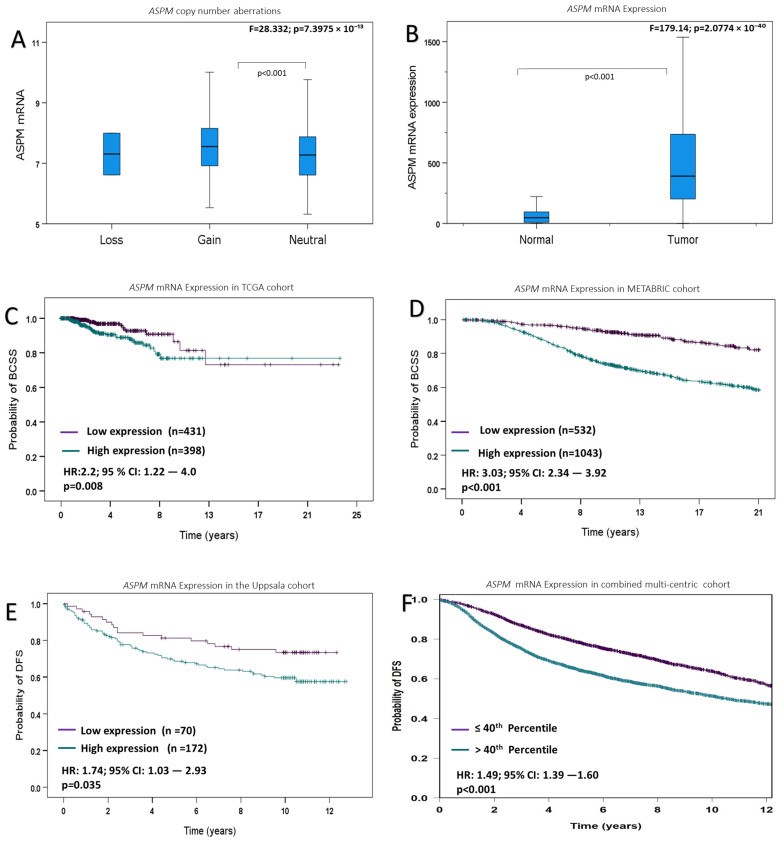
ASPM mRNA expression. (**A**) ASPM mRNA expression and its association with copy number variations. (**B**) ASPM expression in BC and normal tissues in TCGA cohort. (**C**) ASPM mRNA expression against Breast Cancer Specific Survival (BCSS) in TCGA cohort. (**D**) ASPM mRNA expression against BCSS in the METABRIC cohort. (**E**) ASPM mRNA expression against disease-free survival (DFS) in the Uppsala cohort. (**F**) ASPM mRNA expression against DFS in the combined multicentric cohort.

A significant association was observed between high ASPM mRNA expression and ASPM gene CN gain (*p* < 0.001) (Figure 1A).

### 3.3. ASPM mRNA Expression

ASPM mRNA expression levels in BC and matched normal tissues were assessed in the TCGA cohort (Figure 1B); the expression levels of ASPM in BC tissues were significantly higher than those in corresponding normal tissues.

### 3.4. Association of ASPM mRNA Expression with Clinicopathological Parameters

In the TCGA cohort, high ASPM mRNA expression was significantly associated with larger tumor size, higher tumor grade, high mitotic score, LVI, ER positivity, and HER2 negativity (*p* < 0.001). The association between ASPM mRNA and aggressive tumor features including the association with higher tumor grades (*p* < 0.001) were also validated and confirmed in the METABRIC database; these findings are presented in Table 1.

In the Uppsala cohort, high ASPM mRNA expression was significantly associated with high tumor grade, high ki67 expression and P53 expression (mutated type). In the combined multicentric cohort, high ASPM expression was significantly associated with a higher grade, higher NPI score, ER negativity, PR negativity, HER2 positive status, P53 mutated types, and basal BC subtypes (Supplementary Figures S2–S5).

### 3.5. Association of ASPM mRNA Expression with the Patient Outcome

The prognostic ability of ASPM mRNA was tested in four international cohorts.

In the TCGA cohort, high ASPM mRNA expression was associated with shorter survival (*p* = 0.008). Similar findings were observed in the METABRIC cohort, where there was an association between high ASPM expression and poor patient outcomes (*p* < 0.001). In the Uppsala cohort, high ASPM mRNA expression was associated with shorter survival (*p* = 0.036). Similarly, in the combined multicentric cohort, high ASPM expression was significantly associated with shorter BC survival (*p* < 0.001) (Figure 1C–F).

**Figure 2:**
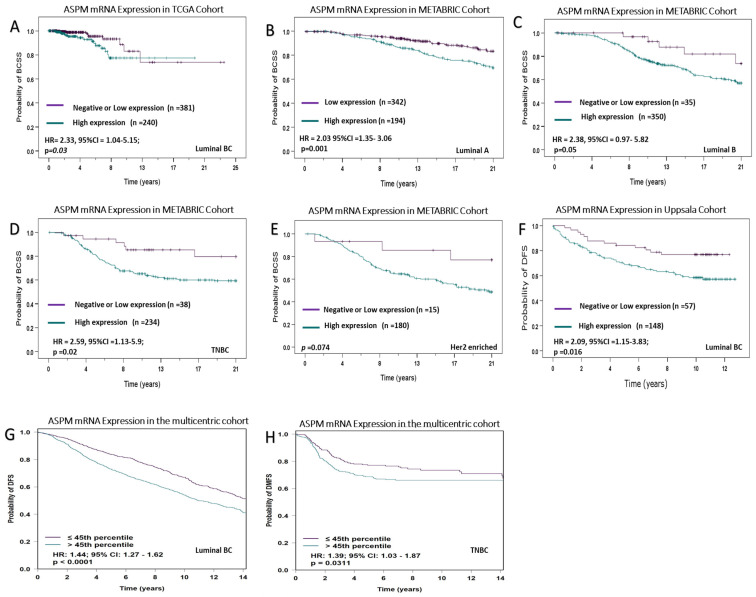
ASPM mRNA expression and survival outcomes. (**A**) ASPM mRNA expression against breast cancer-specific survival (BCSS) in luminal BC in the TCGA cohort. (**B**) ASPM mRNA expression against BCSS in luminal A subtype in the METABRIC cohort. (**C**) ASPM mRNA expression against BCSS in luminal B subtype in the METABRIC cohort. (**D**) ASPM mRNA expression against BCSS in TNBC subtype in the METABRIC cohort. (**E**) ASPM mRNA expression against BCSS in Her2 enriched BC subtype in the METABRIC cohort. (**F**) ASPM mRNA expression against disease-free survival (DFS) in luminal BC in the Uppsala cohort. (**G**) ASPM mRNA expression against DFS in luminal BC in the combined multicentric cohort. (**H**) ASPM mRNA expression against DFS in TNBC in the combined multicentric cohort.

When the cohort was stratified based on the molecular subtypes, high ASPM mRNA expression in the TCGA cohort was predictive of shorter survival in luminal BC (*p* = 0.03). Similar associations were observed in the METABRIC cohort, where ASPM was predictive of BCSS in the luminal A (*p* = 0.001) and TNBC subtypes (*p* = 0.02). However, it was associated with borderline poor survival in the luminal B subtype (*p* = 0.05). In the Uppsala cohort, high ASPM mRNA expression was predictive of shorter BCSS in luminal BC (*p* = 0.016). Similarly, in the multicentric combined cohort, high ASPM mRNA expression was predictive of poor outcome in the luminal (*p* = 0.001) and TNBC (*p* = 0.03) subtypes (Figure 2).

In a multivariate Cox regression analysis, high ASPM mRNA expression remained an independent predictor of poor survival in the METABRIC cohort (*p* < 0.001). When added to other prognostic covariates, similar results were observed in the TCGA cohort (*p* = 0.04) and Uppsala cohort (*p* = 0.03), Table 2.

### 3.6. ASPM Protein Expression

**Figure 3:**
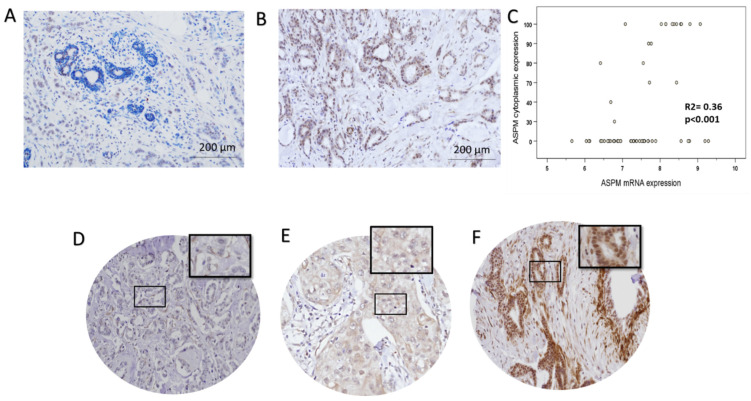
Immunohistochemical analysis of ASPM expression. (**A**) Immunohistochemical (IHC) analysis of ASPM in full-face sections reveals negative immunoreactivity in the normal terminal duct-lobular units (magnification: ×200). (**B**) ASPM expression is high in invasive breast cancer cells compared to normal epithelial cells (magnification: ×200). (**C**) ASPM cytoplasmic expression and its corresponding mRNA expression. (**D**) Negative ASPM IHC expression (magnification: ×200). (**E**) Weak ASPM IHC expression in invasive breast cancer TMA cores (magnification: ×200). (**F**) High ASPM IHC expression in invasive breast cancer TMA cores (magnification: ×200).

On the protein level, ASPM expression was detected in the cytoplasm of invasive BC cells, with occasional nuclear expression, showing various intensities from weak to strong with a homogenous distribution pattern. Epithelial cells in the adjacent normal breast TDLUs showed negative staining of ASPM (Figure 3A). After the exclusion of uninformative cases (i.e., lost, folded or cores containing scanty tumor cells <15%), a total of 1300 tissue cores were included in the analysis. 157/1300 cases (12%) showed high cytoplasmic ASPM expression (Figure 3F). A high interobserver (ICC = 0.72, *p* < 0.001) and intra-observer (ICC = 0.78, *p* < 0.001) concordance was observed.

### 3.7. Association of ASPM Protein Expression with Clinicopathological Parameters

High cytoplasmic expression of ASPM protein was significantly associated with higher tumor grade, elevated mitotic scores, increased Ki67 labeling index, pronounced nuclear pleomorphism, and poor Nottingham Prognostic Index (NPI) (*p* < 0.001); (Table 3).

### 3.8. Association of ASPM Protein Expression with the Patient Outcome

**Figure 4:**
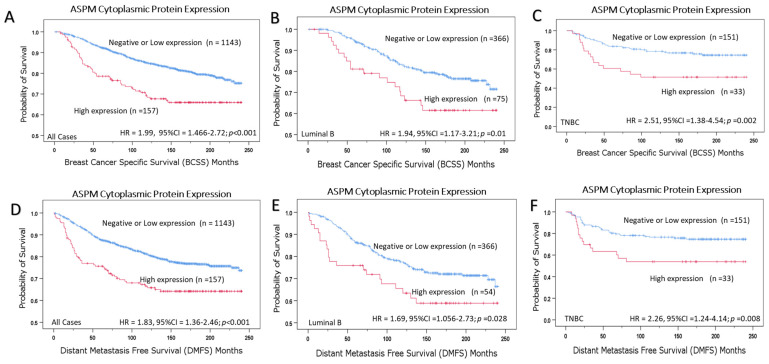
Survival analysis of ASPM cytoplasmic expression. Kaplan–Meier survival plots showing the association between ASPM cytoplasmic expression and BCSS in (**A**) All cohort, (**B**) Luminal B subtype, and (**C**) TNBC, in addition to the association between ASPM cytoplasmic expression and DMFS in (**D**) All cohort, (**E**) Luminal B subtype, and (**F**) TNBC.

Patients with high ASPM cytoplasmic expression demonstrated significantly shorter survival in terms of BCSS and DMFS compared to those with low expression (*p* < 0.001). When the cohort was stratified according to the molecular subtypes, a similar association was observed in the luminal B subtype (*p* = 0.01 and *p* = 0.028) as well as TNBC (*p* = 0.002 and *p* = 0.008) for BCSS and DMFS respectively; (Figure 4).

In multivariate Cox regression models, high cytoplasmic ASPM expression remained an independent predictor of shorter BCSS (*p* = 0.007) and DMFS (*p* = 0.022) when considering other prognostic covariates such as nodal stage, tumor grade, mitosis score, and Ki67 index (Table 4).

### 3.9. ASPM mRNA and Protein Levels

In the METABRIC-Nottingham samples that have data on both mRNA and protein expression, there was a trend toward a positive correlation between mRNA and cytoplasmic expression for ASPM (r = 0.36, *p* < 0.001) (Figure 3C).

### 3.10. Correlation Between ASPM and Ki67 Expression

A highly significant positive correlation was detected between the expression levels of ASPM and Ki67 at the mRNA level in both the METABRIC dataset (r = 0.73, *p* < 0.001) and the TCGA dataset (r = 0.77, *p* < 0.001), in addition to the combined multi-centric cohort (r = 0.89, *p* < 0.001). However, a weaker yet significant correlation has been observed between ASPM and Ki67 expression in the Uppsala cohort (r = 0.24, *p* < 0.001). Furthermore, consistent results were observed at the protein level, between cytoplasmic ASPM and Ki67 (r = 0.113, *p* < 0.001) (Supplementary Figure S6). These findings provide evidence supporting the role of ASPM in tumor cell proliferation and growth.

### 3.11. Correlation Between ASPM Expression and Drug Treatment

**Figure 5:**
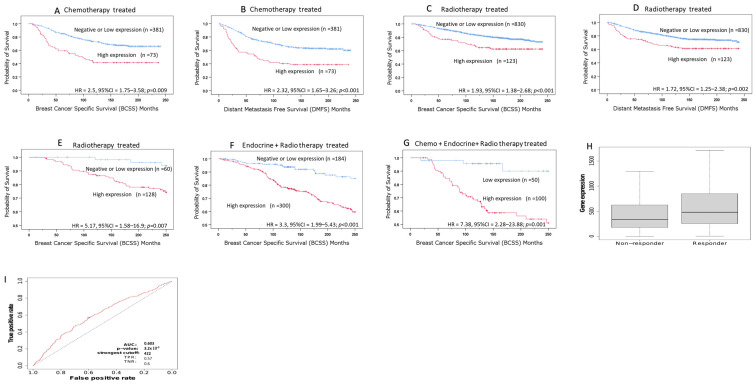
Survival Analysis of ASPM expression in chemotherapy and radiotherapy-treated patients. Kaplan–Meier survival plots showing the association between ASPM cytoplasmic expression and (**A**) BCSS in chemotherapy-treated patients, (**B**) DMFS in chemotherapy-treated patients, (**C**) BCSS in radiotherapy-treated patients and (**D**) DMFS in radiotherapy-treated patients in Nottingham cohort, in addition to the association between ASPM mRNA expression and BCSS in patients receiving (**E**) radiotherapy, (**F**) radiotherapy and hormonal therapy, and (**G**) radiotherapy, hormonal therapy and chemotherapy, in the METABRIC cohort, (**H**) Patients who responded to taxane treatment had significantly higher ASPM levels than non-responders (**I**) Receiver operating characteristic (ROC) curve showing the predictive performance, with an area under the curve (AUC) of 0.603, and a *p*-value 3.2 × 10^−9^.

We stratified our cohorts based on adjuvant treatment to examine its impact on outcomes. We analyzed two cohorts, the METABRIC and Nottingham cohorts, to evaluate the predictive value of ASPM at both mRNA and protein levels. In patients receiving chemotherapy, high ASPM cytoplasmic expression was predictive of unfavorable outcomes, as indicated by significantly shorter BCSS (*p* = 0.009) and DMFS (*p* < 0.001). Similar associations were also observed in patients who underwent radiotherapy where high ASPM cytoplasmic expression maintained its correlation with poorer BCSS (*p* < 0.001) and DMFS (*p* = 0.002); (Figure 5A–D). However, untreated patients did not show such associations.

Furthermore, in the METABRIC cohort at the mRNA level, high ASPM expression was predictive of poor survival in patients receiving radiotherapy alone (*p* = 0.007), radiotherapy combined with hormonal therapy (*p* < 0.001) and radiotherapy, combined with hormonal and chemotherapy (*p* = 0.001); (Figure 5E–G).

To further validate the role of ASPM as a predictive biomarker, we analyzed the correlation between ASPM gene expression and the response to taxane treatment, as well as clinical outcomes in patients. This evaluation was conducted using the ROC plotter (https://www.rocplot.com/site/treatment) accessed on 20 March 2024. This revealed that patients who responded to taxane exhibited significantly higher levels of ASPM compared to those who did not, (Figure 5H,I), suggesting that ASPM may provide a predictive indication in BC treatment.

## 4. Discussion

Targeting proteins involved in mitotic cell division is an effective way to monitor and control tumor growth and progression [46]. ASPM was identified as one of the significantly upregulated genes following the analysis of TCGA images with high mitotic scores, which was further supported by a recent analysis of proteins from purified human mitotic spindles [47].

ASPM is a known regulator of mitosis, specifically involved in spindle organization and microtubule assembly [48,49], and plays a critical role in mitotic spindle regulation [50], promoting cell proliferation and regulating the cell cycle, especially during the late G1 checkpoint [51]. Furthermore, ASPM controls the proliferative symmetry of progenitor cells [52], ensuring proper cell division. Mutations in ASPM may affect the fidelity of chromosome segregation and the plane of cell division.

Emerging evidence suggests that ASPM is involved in the regulation of cancer cell stemness. Notably, recent findings by Tsai’s group [53] demonstrated that ASPM enhances a stem cell phenotype in prostate cancer cells by augmenting Wnt/β-catenin signaling, thereby promoting tumor aggressiveness. Similar effects of ASPM have been reported in pancreatic adenocarcinoma and other malignancies, indicating its role in inducing cancer cell stemness and accelerating tumor progression [54]. Considering these findings, ASPM has gained attention as a potential therapeutic target with implications for future interventions.

One of the aims of this study is to investigate the protein expression of ASPM in BC tissues. The results demonstrated that ASPM was detectable in the cytoplasm of tumor cells. While there is limited information available regarding its subcellular localization and functions in specific cellular compartments, some studies have provided insight into its roles. A study by Xie et al. [55] found that the ASPM protein was primarily localized in the cytoplasm of prostatic cancer cells. In human cell culture studies, it was observed that ASPM has specific cellular localization patterns during different stages of the cell cycle. During interphase, ASPM is located at the centrosome, positioned at the centre of the cell, and tightly associated with the nuclear envelope. However, during mitosis, ASPM localizes to the spindle poles from prophase through telophase [20,56], indicating a dynamic change in localization during the cell cycle and their functional role in cell division. A study by Hsu et al. [57] investigated different isoforms of ASPM and their functions in cancer cells. They found that ASPM-iI is predominantly localized in the cytoplasm of tumor cells and interacts with Dvl-2, a member of the Wnt signaling pathway, promoting stemness and aggressiveness.

Both at the mRNA level in the TCGA cohort and the protein level in the Nottingham cohort, the expression levels of ASPM were found to be significantly higher in BC tissues compared to their corresponding normal tissues. This observation is supported by the findings of Xie et al. [55] who reported that ASPM protein was primarily localized in prostatic cancer cells with weak or negative expression in adjacent non-cancerous tissues.

According to our findings, high ASPM expression at mRNA and protein levels was significantly correlated with proliferation, as evaluated using the Ki67 labelling index, confirming that it is critical for proliferation and progression.

We revealed a significant association between high ASPM mRNA and protein expression, with poor clinicopathological parameters including high tumor grade, high mitotic score, and NPI.

A recent bioinformatic study conducted on BC reported that the overexpression of ASPM is related to aggressive malignant features, such as high tumor grade and advanced stage [58], whereas its downregulation suppresses tumor cell proliferation, invasion, migration, and epithelial-to-mesenchymal transition [15,16,59,60].

These results are aligned with Bikeye et al. who state that ASPM expression in glioma is positively correlated with tumor grade and associated with tumor recurrence, while silencing of ASPM results in dramatic proliferation arrest and cell death in glioma sphere models [12]. A study on ovarian cancer also revealed a statistically significant correlation between ASPM and tumor grade [61].

In medulloblastoma, ASPM is overexpressed and its knockdown inhibits both tumor and stem cell proliferation [62].

Our results suggest that ASPM expressions have prognostic implications in BC. High cytoplasmic expression is associated with poorer survival, particularly in the luminal B and TNBC subtypes. These findings suggest that the ASPM protein expression has implications for patient survival. Furthermore, at the mRNA level, we detected a significant correlation between high ASPM and short patient survival, where high ASPM mRNA predicted poor outcomes in the luminal and TNBC subtypes. These findings suggest that ASPM is involved in tumorigenic pathways and may serve as a marker of poor prognosis in luminal and triple-negative breast cancer (TNBC) subtypes. Therefore, ASPM has the potential to be utilized as an additional marker for progression and transformation in these cancer types.

ASPM has been consistently associated with worse patient survival rates in various types of cancer. In HCC, for example, ASPM was strongly linked to reduced overall survival. The high expression of ASPM was an independent predictor of poor overall survival in endometrial carcinoma [60] as well as poor overall survival and recurrence-free survival in lung adenocarcinoma [63].

Our study revealed that ASPM is an independent prognostic marker significantly associated with shorter DMFS and BCSS, even when considering other prognostic factors such as the Ki67 index in a Cox regression model. Additionally, ASPM demonstrated superior predictive ability compared to Ki67. These findings highlight the potential clinical relevance of ASPM as a powerful biomarker for improving survival rate prediction and prognostic stratification in BC.

Since the high expression of ASPM was associated with worse prognostic features and outcomes and, given that it is linked to tumor cell proliferation, we hypothesized that ASPM overexpression could play a significant role in resistance to radiotherapy and chemotherapy. We demonstrated that patients with high ASPM expression showed poorer outcomes when radiotherapy and chemotherapy were received.

Recent studies found that reducing the expression of ASPM enhanced the sensitivity of human cells to radiation and other DNA-damaging agents [64]. The knockdown of ASPM impaired the repair of DNA double-strand breaks (DSBs) and increased the formation of chromosomal aberration [64]. A recent study reported that the ASPM gene plays a new role in repairing DNA breaks as it helps in stabilizing BRCA1, which is involved in DNA repair, thus improving the cell’s ability to fix breaks. When ASPM is inhibited, BRCA1 becomes unstable, leading to compromised DNA repair [65].

These findings suggest that ASPM plays a role in DNA repair, particularly in the non-homologous end joining (NHEJ) pathway, and reducing its expression could be a potential strategy to improve tumor cell killing in radiation therapy.

Jinming Yu et al. [66] found that ASPM is highly expressed in radiation-resistant samples of lung adenocarcinoma. This high expression correlated with disease advancement and enabled radiation-resistant cells to bypass the spindle checkpoint and complete cell division after irradiation by destabilizing microtubules, resulting in increased chromosome mis-segregation and chromosomal instability during mitosis.

Patients who exhibited a positive response to taxane treatment demonstrated notably elevated levels of ASPM compared to those who did not respond. This observation suggests that ASPM may serve not only as a prognostic marker but also as a potential predictive indicator for the efficacy of taxane medications in the treatment of BC.

These results indicate that ASPM could potentially contribute to treatment resistance and that assessment of ASPM expression before treatment could predict treatment response and resistance. These results indicate that targeting ASPM could be a promising approach to developing novel cancer therapies.

## 5. Conclusions

In conclusion, recent research in different malignant tumors revealed that ASPM is highly expressed in a wide range of human malignancies and is linked to aggressive malignant features, worse outcomes, and recurrence. Sufficient evidence revealed that ASPM can be considered a promising genetic marker and a therapeutic target [20].

We believe that targeting ASPM may provide a novel and alternative strategy to inhibit BC cell growth and progression. This study has been conducted on a large number of BC cases and demonstrates that ASPM may serve as a novel prognostic marker and therapeutic target for BC. Further functional studies of ASPM with consideration of its subcellular localization in tumor cells are warranted.

## Figures and Tables

**Table 1:** The association between ASPM mRNA expression and clinicopathological parameters in the Molecular Taxonomy of Breast Cancer International Consortium (METABRIC) and in the Cancer Genome Atlas (TCGA) breast cancer series.

Parameter	METABRIC ASPM mRNA			TCGA ASPM mRNA		
	Low No (%)	High No (%)	x^2^ *p*-Value	Low No (%)	High No (%)	x^2^ *p*-Value
Patient age (years) <50 ≥50	112 (16.5) 568 (83.5)	271 (21.5) 988 (78.5)	7.116 0.008	108 (24.5) 333 (75.5)	123 (29.8) 290 (70.2)	3.027 0.082
Tumor size <2 ≥2	264 (38.4) 424 (61.6)	358 (28.3) 907 (71.7)	20.827 <0.001	91 (30.0) 212 (70.0)	60 (19.5) 247 (80.5)	9.007 0.003
Tumor grade 1 2 3	120 (18.7) 364 (56.6) 159 (24.7)	50 (4.0) 406 (32.5) 793 (63.5)	288.873 <0.001	67 (38.3) 89 (50.9) 19 (10.9)	12 (5.9) 56 (27.5) 136 (66.7)	132.675 <0.001
Molecular subtypes Luminal A Luminal B Basal-like HER2 enriched. Normal	459 (66.1) 49 (7.1) 19 (2.7) 20 (2.9) 147 (21.2)	259 (20.1) 439 (34.1) 310 (24.1) 220 (17.1) 52 (4.0)	726.897 <0.001	313 (71.0) 25 (5.7) 7 (1.6) 15 (3.4) 26 (5.9)	92 (22.3) 116 (28.1) 126 (30.5) 41 (9.9) 4 (1.0)	318.383 <0.001
Mitotic score 1 2 3	N/A	N/A	N/A	199 (81.9) 29 (11.9) 15 (6.2)	74 (33.6) 39 (17.7) 107 (48.6)	127.254 <0.001
Histological subtypes Invasive duct carcinoma (NST) Invasive lobular carcinoma Mixed NST and special type Other special types *	465 (69.9) 75 (11.3) 47 (7.1) 76 (11.5) 2 (0.3)	1079 (85.6) 72 (5.7) 43 (3.4) 37 (2.9) 30 (2.4)	103.317 <0.001	274 (62.1) 128 (29.0) 9 (2.0) 30 (6.9)	339 (82.0) 49 (11.9) 7 (1.7) 18 (4.4)	52.294 <0.001
Lymph nodal stage 1 2 3	394 (57.0) 209 (30.2) 88 (12.7)	641 (50.0) 413 (32.2) 228 (17.8)	11.917 0.003	101 (22.9) 237 (53.7) 103 (23.4)	61 (14.8) 262 (63.4) 90 (21.8)	11.099 0.005
Nottingham Prognostic Index Good Moderate Poor	362 (52.2) 299 (43.1) 33 (4.8)	318 (24.7) 802 (62.4) 166 (12.9)	158.723 <0.001	N/A	N/A	N/A
Lympho-vascular invasion Negative Positive	158 (23.2) 522 (76.8)	578 (45.8) 685 (54.2)	95.430 <0.001	312 (70.7) 129 (29.3)	247 (59.8) 166 (40.2)	11.293 <0.001
Oestrogen receptor Negative Positive	64 (9.2) 630 (90.8)	410 (31.9) 876 (68.1)	127.110 <0.001	38 (8.9) 390 (91.1)	147 (37.1) 249 (62.9)	94.234 <0.001
Progesterone receptor Negative Positive	218 (31.4) 476 (68.6)	722 (56.1) 564 (43.9)	110.557 <0.001	81 (19.0) 346 (81.0)	191 (48.2) 205 (51.8)	79.512 <0.001
HER2 status Negative Positive	670 (96.5) 24 (3.5)	1063 (82.7) 223 (17.3)	79.561 <0.001	387 (88.0) 53 (12.0)	337 (81.6 76 (18.4)	6.919 0.031
Triple-negative status Non-triple negative Triple-negative	644 (92.8) 50 (7.2)	1016 (79.0) 270 (21.0)	63.268 <0.001	430 (97.5) 11 (2.5)	329 (79.6) 84 (20.3)	68.715 <0.001
P53 mutation Status Mutation Wild type	11 (1.6) 275 (39.6)	88 (6.8) 446 (34.7)	27.956 <0.001	N/A	N/A	N/A
Ki67 index Low High	604 (98.1) 12 (1.9)	359 (38.1) 583 (61.9)	566.907 <0.001	356 (80.7) 85 (19.3)	237 (57.4) 176 (42.6)	54.749 <0.001

* Other special types: Mucinous, tubular and papillary. *p* value < 0.05 significant. ASPM: Assembly factor for spindle microtubules. NST: No-special types. N/A: Not Applicable.

**Table 2:** The Multivariate Cox regression analysis results for predictors of breast-cancer-specific survival (BCSS) in (A) METABRIC cohort, (B) TCGA cohort, (C) Uppsala cohort.

	Model Parameters	Breast Cancer Specific Survival (BCSS)		
		HR	95% (CI)	*p*-Value
(A)	*ASPM* mRNA	2.031	1.398–2.950	<0.001
	Nodal stage	1.96	1.694–2.268	<0.001
	Tumor grade	1.304	1.050–1.620	0.016
	Ki67 score	1.538	1.166–2.029	0.002
(B)	*ASPM* mRNA	1.856	1.005–3.428	0.04
	Nodal stage	2.661	1.688–4.196	<0.001
	Ki67 score	2.131	1.201–3.782	0.01
(C)	*ASPM* mRNA	1.776	1.053–2.996	0.031
	ER status.	0.816	0.442–1.505	0.514
	Lymph node status	1.91	1.234–2.957	0.004

*p* value < 0.05 significant.

**Table 3:** The association between ASPM cytoplasmic protein expression and clinicopathological parameters in Nottingham cohort.

Parameter	ASPM Cytoplasmic Expression		
	Low No (%)	High No (%)	x^2^ *p*-Value
Patient age (years) <50 ≥50	346 (30.3) 797 (69.7)	61 (38.9) 96 (61.1)	4.728 0.030
Tumor size (cm) <2 ≥2	712 (62.3) 431 (37.7)	84 (53.5) 73 (46.5)	4.492 0.034
Tumor grade 1 2 3	181 (15.8) 461 (40.3) 501 (43.8)	20 (12.7) 41 (26.1) 96 (61.1)	17.094 <0.001
Tubule formation 1 2 3	89 (7.8) 320 (28.0) 734 (64.2)	10 (6.4) 49 (31.2) 98 (62.4)	0.936 0.626
Mitotic score 1 2 3	565 (49.4) 226 (19.8) 352 (30.8)	50 (31.8) 38 (24.2) 69 (43.9)	17.731 <0.001
Nuclear pleomorphism 1 2 3	16 (1.4) 348 (30.4) 779 (68.2)	2 (1.3) 22 (14.0) 133 (84.7)	18.509 <0.001
Molecular subtypes Luminal A Luminal B HER2 Triple negative breast cancer	428 (43.1) 366 (36.9) 47 (4.7) 151 (15.2)	39 (27.3) 54 (37.8) 17 (11.9) 33 (23.1)	23.767 <0.001
Histological subtypes Non-specific type (NST) Lobular Mixed NST and special type Other special types *	718 (62.8) 105 (9.2) 267 (23.4) 53 (4.6)	122 (77.7) 4 (2.5) 25 (15.9) 6 (3.8)	16.089 0.003
Axillary nodal stage 1 2 3	718 (62.8) 310 (27.1) 115 (10.1)	81 (51.6) 53 (33.8) 23 (14.6)	7.745 0.021
Nottingham Prognostic Index Good Moderate Poor	403 (35.3) 558 (48.8) 182 (15.9)	37 (23.6) 82 (52.2) 38 (24.2)	11.494 0.003
Lympho-vascular invasion Negative Positive	820 (71.7) 323 (28.3)	108 (68.8) 49 (31.2)	0.589 0.443
Oestrogen receptor Negative Positive	209 (18.3) 934 (81.7)	51 (32.7) 105 (67.3)	17.797 <0.001
Progesterone receptor Negative Positive	453 (39.8) 684 (60.2)	82 (52.9) 73 (47.1)	9.592 0.002
HER2 status Negative Positive	1000 (87.5) 143 (12.5)	118 (75.6) 38 (24.4)	16.068 <0.001
Ki67 index Low High	469 (54.5) 392 (45.5)	42 (38.5) 67 (61.5)	9.861 0.002

* Other special types. Medullary, mucinous, tubular. *p* value < 0.05 significant. ASPM: Abnormal spindle-like microcephaly-associated. HER2 final status is achieved using combination of IHC and chromogenic in situ hybridization (CISH).

**Table 4:** Multivariate Cox regression analysis results for predictors of breast-cancer-specific survival and distant metastasis free survival in the Nottingham cohort.

Model Parameters	Breast Cancer Specific Survival (BCSS)			Distant Metastasis Free Survival (DMFS)		
	HR	95% (CI)	*p*-Value	HR	95% (CI)	*p*-Value
ASPM cytoplasmic expression	1.702	1.154–2.510	0.007	1.545	1.063–2.246	0.022
Nodal stage	2.285	1.812–2.882	<0.001	2.147	1.722–2.677	<0.001
Tumor grade	2.413	1.506–3.865	<0.001	1.925	1.267–2.926	0.002
Mitosis score	0.937	0.678–1.295	0.694	0.938	0.695–1.264	0.673
Ki67 score	1.122	0.774–1.628	0.543	1.333	0.939–1.894	0.108

*p* value < 0.05 significant.

## Data Availability

The authors confirm the data that have been used in this work are available upon reasonable request.

## Supplementary Materials

The following supporting information can be downloaded at: https://www.mdpi.com/article/10.3390/cancers16223814/s1, Table S1. list of all the datasets used to construct the multicentre combined cohort to investigate the expression of *ASPM* transcript (*n* = 7252) in early-stage breast cancer patients using bc-GenExMine; Table S2. list of all the datasets used to construct the multicenter combined cohort with different microarrays platforms used (using bc-GenExMiner); Table S3. Preprocessing steps for multicenter combined cohort with different data types using bc-GenExMiner; Table S4. The REMARK checklist; Table S5. Genes enriched in the cell division using Gene Ontology (GO) analysis of DAVID online tool. Figure S1: Identification of Differentially Expressed Genes (DEGs) and Hub Genes in Breast Cancer. Figure S2: ASPM mRNA expression and its correlation with clinicopathological parameters and molecular subtypes in the METABRIC cohort. Figure S3: ASPM mRNA expression and its correlation with clinicopathological parameters and molecular subtypes in the TCGA cohort. Figure S4: ASPM mRNA expression and its correlation with clinicopathological parameters and molecular subtypes in the Uppsala Cohort. Figure S5: ASPM mRNA expression and its correlation with clinicopathological parameters and molecular subtypes in the in the Combined Multi-Centric Cohort. Figure S6. Correlation Between ASPM Expression and Ki67 Levels Across Various Cohorts.

## References

[1] Gonzalez-Angulo A.M., Morales-Vasquez F., Hortobagyi G.N. (2007). Overview of resistance to systemic therapy in patients with breast cancer. Adv. Exp. Med. Biol..

[2] Mc Gee M.M. (2015). Targeting the Mitotic Catastrophe Signaling Pathway in Cancer. Mediat. Inflamm..

[3] Shahriyari L., Abdel-Rahman M., Cebulla C. (2019). BAP1 expression is prognostic in breast and uveal melanoma but not colon cancer and is highly positively correlated with RBM15B and USP19. PLoS ONE.

[4] Lou Y., Fallah Y., Yamane K., Berg P.E. (2018). BP1, a potential biomarker for breast cancer prognosis. Biomark. Med..

[5] Majidinia M., Yousefi B. (2017). DNA repair and damage pathways in breast cancer development and therapy. DNA Repair.

[6] Chan K.S., Koh C.G., Li H.Y. (2012). Mitosis-targeted anti-cancer therapies: Where they stand. Cell Death Dis..

[7] Serrano-del Valle A., Reina-Ortiz C., Benedi A., Anel A., Naval J., Marzo I. (2021). Future prospects for mitosis-targeted antitumor therapies. Biochem. Pharmacol..

[8] Mahalmani V., Sinha S., Prakash A., Medhi B. (2022). Translational research: Bridging the gap between preclinical and clinical research. Indian J. Pharmacol..

[9] Arai T., Okato A., Kojima S., Idichi T., Koshizuka K., Kurozumi A., Kato M., Yamazaki K., Ishida Y., Naya Y. (2017). Regulation of spindle and kinetochore-associated protein 1 by antitumor miR-10a-5p in renal cell carcinoma. Cancer Sci..

[10] Zhang Y., Wang Y., Wei Y., Wu J., Zhang P., Shen S., Saiyin H., Wumaier R., Yang X., Wang C. (2016). Molecular chaperone CCT3 supports proper mitotic progression and cell proliferation in hepatocellular carcinoma cells. Cancer Lett..

[11] Ibrahim A., Toss M.S., Alsaleem M., Makhlouf S., Atallah N., Green A.R., Rakha E.A. (2023). Novel 2 Gene Signatures Associated With Breast Cancer Proliferation: Insights From Predictive Differential Gene Expression Analysis. Mod. Pathol..

[12] Bikeye S.-N.N., Colin C., Marie Y., Vampouille R., Ravassard P., Rousseau A., Boisselier B., Idbaih A., Calvo C.F., Leuraud P. (2010). ASPM-associated stem cell proliferation is involved in malignant progression of gliomas and constitutes an attractive therapeutic target. Cancer Cell Int..

[13] Buchman J.J., Durak O., Tsai L.-H. (2011). ASPM regulates Wnt signaling pathway activity in the developing brain. Genes Dev..

[14] Major M.B., Roberts B.S., Berndt J.D., Marine S., Anastas J., Chung N., Ferrer M., Yi X., Stoick-Cooper C.L., Von Haller P.D. (2008). New regulators of Wnt/β-catenin signaling revealed by integrative molecular screening. Sci. Signal..

[15] Lin S.-Y., Pan H.-W., Liu S.-H., Jeng Y.-M., Hu F.-C., Peng S.-Y., Lai P.-L., Hsu H.-C. (2008). ASPM is a novel marker for vascular invasion, early recurrence, and poor prognosis of hepatocellular carcinoma. Clin. Cancer Res..

[16] Drozdov I., Bornschein J., Wex T., Valeyev N.V., Tsoka S., Malfertheiner P. (2012). Functional and topological properties in hepatocellular carcinoma transcriptome. PLoS ONE.

[17] Jung H.M., Choi S.J., Kim J.K. (2009). Expression profiles of SV40-immortalization-associated genes upregulated in various human cancers. J. Cell. Biochem..

[18] Hagemann C., Anacker J., Gerngras S., Kühnel S., Said H.M., Patel R., Kämmerer U., Vordermark D., Roosen K., Vince G.H. (2008). Expression analysis of the autosomal recessive primary microcephaly genes MCPH1 (microcephalin) and MCPH5 (ASPM, abnormal spindle-like, microcephaly associated) in human malignant gliomas. Oncol. Rep..

[19] Horvath S., Zhang B., Carlson M., Lu K., Zhu S., Felciano R., Laurance M., Zhao W., Qi S., Chen Z. (2006). Analysis of oncogenic signaling networks in glioblastoma identifies ASPM as a molecular target. Proc. Natl. Acad. Sci. USA.

[20] Kouprina N., Pavlicek A., Collins N.K., Nakano M., Noskov V.N., Ohzeki J., Mochida G.H., Risinger J.I., Goldsmith P., Gunsior M. (2005). The microcephaly ASPM gene is expressed in proliferating tissues and encodes for a mitotic spindle protein. Hum. Mol. Genet..

[21] Peyre M., Commo F., Dantas-Barbosa C., Andreiuolo F., Puget S., Lacroix L., Drusch F., Scott V., Varlet P., Mauguen A. (2010). Portrait of ependymoma recurrence in children: Biomarkers of tumor progression identified by dual-color microarray-based gene expression analysis. PLoS ONE.

[22] Tomczak K., Czerwińska P., Wiznerowicz M. (2015). The Cancer Genome Atlas (TCGA): An immeasurable source of knowledge. Contemp. Oncol..

[23] Ibrahim A., Lashen A.G., Katayama A., Mihai R., Ball G., Toss M.S., Rakha E.A. (2022). Defining the area of mitoses counting in invasive breast cancer using whole slide image. Mod. Pathol..

[24] Hoadley K.A., Yau C., Wolf D.M., Cherniack A.D., Tamborero D., Ng S., Leiserson M.D.M., Niu B., McLellan M.D., Uzunangelov V. (2014). Multiplatform analysis of 12 cancer types reveals molecular classification within and across tissues of origin. Cell.

[25] Wang K., Singh D., Zeng Z., Coleman S.J., Huang Y., Savich G.L., He X., Mieczkowski P., Grimm S.A., Perou C.M. (2010). MapSplice: Accurate mapping of RNA-seq reads for splice junction discovery. Nucleic Acids Res..

[26] Li B., Dewey C.N. (2011). RSEM: Accurate transcript quantification from RNA-Seq data with or without a reference genome. BMC Bioinform..

[27] Curtis C., Shah S.P., Chin S.-F., Turashvili G., Rueda O.M., Dunning M.J., Speed D., Lynch A.G., Samarajiwa S., Yuan Y. (2012). The genomic and transcriptomic architecture of 2,000 breast tumours reveals novel subgroups. Nature.

[28] Ivshina A.V., George J., Senko O., Mow B., Putti T.C., Smeds J., Lindahl T., Pawitan Y., Hall P., Nordgren H. (2006). Genetic reclassification of histologic grade delineates new clinical subtypes of breast cancer. Cancer Res..

[29] Pawitan Y., Bjöhle J., Amler L., Borg A.-L., Egyhazi S., Hall P., Han X., Holmberg L., Huang F., Klaar S. (2005). Gene expression profiling spares early breast cancer patients from adjuvant therapy: Derived and validated in two population-based cohorts. Breast Cancer Res..

[30] Koboldt D., Fulton R., McLellan M., Schmidt H., Kalicki-Veizer J., McMichael J., Fulton L., Dooling D., Ding L., Mardis E. (2012). Comprehensive molecular portraits of human breast tumours. Nature.

[31] Rakha E.A., Martin S., Lee A.H.S., Morgan D., Pharoah P.D.P., Hodi Z., MacMillan D., Ellis I.O. (2012). The prognostic significance of lymphovascular invasion in invasive breast carcinoma. Cancer.

[32] Aleskandarany M.A., Abduljabbar R., Ashankyty I., Elmouna A., Jerjees D., Ali S., Buluwela L., Diez-Rodriguez M., Caldas C., Green A.R. (2016). Prognostic significance of androgen receptor expression in invasive breast cancer: Transcriptomic and protein expression analysis. Breast Cancer Res. Treat..

[33] Rakha E.A., Agarwal D., Green A.R., Ashankyty I., Ellis I.O., Ball G., Alaskandarany M.A. (2017). Prognostic stratification of oestrogen receptor-positive HER2-negative lymph node-negative class of breast cancer. Histopathology.

[34] Rakha E.A., Elsheikh S.E., Aleskandarany M.A., Habashi H.O., Green A.R., Powe D.G., El-Sayed M.E., Benhasouna A., Brunet J.S., Akslen L.A. (2009). Triple-negative breast cancer: Distinguishing between basal and nonbasal subtypes. Clin. Cancer Res..

[35] Muftah A.A., Aleskandarany M.A., Al-Kaabi M.M., Sonbul S.N., Diez-Rodriguez M., Nolan C.C., Caldas C., Ellis I.O., Rakha E.A., Green A.R. (2017). Ki67 expression in invasive breast cancer: The use of tissue microarrays compared with whole tissue sections. Breast Cancer Res. Treat..

[36] Rakha E.A., Pinder S.E., Bartlett J.M., Ibrahim M., Starczynski J., Carder P.J., Provenzano E., Hanby A., Hales S., Lee A.H. (2015). Updated UK Recommendations for HER2 assessment in breast cancer. J. Clin. Pathol..

[37] Hammond M.E., Hayes D.F., Dowsett M., Allred D.C., Hagerty K.L., Badve S., Fitzgibbons P.L., Francis G., Goldstein N.S., Hayes M. (2010). American Society of Clinical Oncology/College Of American Pathologists guideline recommendations for immunohistochemical testing of estrogen and progesterone receptors in breast cancer. J. Clin. Oncol..

[38] Goldhirsch A., Wood W.C., Coates A.S., Gelber R.D., Thürlimann B., Senn H.J. (2011). Strategies for subtypes--dealing with the diversity of breast cancer: Highlights of the St. Gallen International Expert Consensus on the Primary Therapy of Early Breast Cancer 2011. Ann. Oncol..

[39] Shannon P., Markiel A., Ozier O., Baliga N.S., Wang J.T., Ramage D., Amin N., Schwikowski B., Ideker T. (2003). Cytoscape: A software environment for integrated models of biomolecular interaction networks. Genome Res..

[40] Abd El-Rehim D.M., Ball G., Pinder S.E., Rakha E., Paish C., Robertson J.F., Macmillan D., Blamey R.W., Ellis I.O. (2005). High-throughput protein expression analysis using tissue microarray technology of a large well-characterised series identifies biologically distinct classes of breast cancer confirming recent cDNA expression analyses. Int. J. Cancer.

[41] McCarty K.S., McCarty K.S. (1984). Histochemical approaches to steroid receptor analyses. Semin. Diagn. Pathol..

[42] Camp R.L., Dolled-Filhart M., Rimm D.L. (2004). X-tile: A new bio-informatics tool for biomarker assessment and outcome-based cut-point optimization. Clin. Cancer Res..

[43] Polley M.Y., Leung S.C., McShane L.M., Gao D., Hugh J.C., Mastropasqua M.G., Viale G., Zabaglo L.A., Penault-Llorca F., Bartlett J.M. (2013). An international Ki67 reproducibility study. J. Natl. Cancer Inst..

[44] Ahlin C., Aaltonen K., Amini R.-M., Nevanlinna H., Fjällskog M.-L., Blomqvist C. (2007). Ki67 and cyclin A as prognostic factors in early breast cancer. What are the optimal cut-off values?. Histopathology.

[45] McShane L.M., Altman D.G., Sauerbrei W., Taube S.E., Gion M., Clark G.M. (2005). REporting recommendations for tumour MARKer prognostic studies (REMARK). Br. J. Cancer.

[46] Otto T., Sicinski P. (2017). Cell cycle proteins as promising targets in cancer therapy. Nat. Rev. Cancer.

[47] Sauer G., Körner R., Hanisch A., Ries A., Nigg E.A., Silljé H.H. (2005). Proteome analysis of the human mitotic spindle. Mol. Cell Proteom..

[48] Higgins J., Midgley C., Bergh A.M., Bell S.M., Askham J.M., Roberts E., Binns R.K., Sharif S.M., Bennett C., Glover D.M. (2010). Human ASPM participates in spindle organisation, spindle orientation and cytokinesis. BMC Cell Biol..

[49] do Carmo Avides M., Glover D.M. (1999). Abnormal spindle protein, Asp, and the integrity of mitotic centrosomal microtubule organizing centers. Science.

[50] Wakefield J.G., Bonaccorsi S., Gatti M. (2001). The drosophila protein asp is involved in microtubule organization during spindle formation and cytokinesis. J. Cell Biol..

[51] Capecchi M.R., Pozner A. (2015). ASPM regulates symmetric stem cell division by tuning Cyclin E ubiquitination. Nat. Commun..

[52] Chenn A., Walsh C.A. (2003). Increased neuronal production, enlarged forebrains and cytoarchitectural distortions in beta-catenin overexpressing transgenic mice. Cereb. Cortex.

[53] Pai V.C., Hsu C.C., Chan T.S., Liao W.Y., Chuu C.P., Chen W.Y., Li C.R., Lin C.Y., Huang S.P., Chen L.T. (2019). ASPM promotes prostate cancer stemness and progression by augmenting Wnt-Dvl-3-β-catenin signaling. Oncogene.

[54] Wang W.Y., Hsu C.C., Wang T.Y., Li C.R., Hou Y.C., Chu J.M., Lee C.T., Liu M.S., Su J.J., Jian K.Y. (2013). A gene expression signature of epithelial tubulogenesis and a role for ASPM in pancreatic tumor progression. Gastroenterology.

[55] Xie J.J., Zhuo Y.J., Zheng Y., Mo R.J., Liu Z.Z., Li B.W., Cai Z.D., Zhu X.J., Liang Y.X., He H.C. (2017). High expression of ASPM correlates with tumor progression and predicts poor outcome in patients with prostate cancer. Int. Urol. Nephrol..

[56] Zhong X., Liu L., Zhao A., Pfeifer G.P., Xu X. (2005). The Abnormal Spindle-like, Microcephaly-associated (ASPM) Gene Encodes a Centrosomal Protein. Cell Cycle.

[57] Hsu C.C., Liao W.Y., Chan T.S., Chen W.Y., Lee C.T., Shan Y.S., Huang P.J., Hou Y.C., Li C.R., Tsai K.K. (2019). The differential distributions of ASPM isoforms and their roles in Wnt signaling, cell cycle progression, and pancreatic cancer prognosis. J. Pathol..

[58] Tang J., Lu M., Cui Q., Zhang D., Kong D., Liao X., Ren J., Gong Y., Wu G. (2019). Overexpression of ASPM, CDC20, and TTK Confer a Poorer Prognosis in Breast Cancer Identified by Gene Co-expression Network Analysis. Front. Oncol..

[59] Zhang H., Yang X., Zhu L., Li Z., Zuo P., Wang P., Feng J., Mi Y., Zhang C., Xu Y. (2021). ASPM promotes hepatocellular carcinoma progression by activating Wnt/β-catenin signaling through antagonizing autophagy-mediated Dvl2 degradation. FEBS Open Bio..

[60] Zhou J.W., Wang H., Sun W., Han N.N., Chen L. (2020). ASPM is a predictor of overall survival and has therapeutic potential in endometrial cancer. Am. J. Transl. Res..

[61] Brüning-Richardson A., Bond J., Alsiary R., Richardson J., Cairns D., McCormack L., Hutson R., Burns P., Wilkinson N., Hall G. (2011). ASPM and microcephalin expression in epithelial ovarian cancer correlates with tumour grade and survival. Br. J. Cancer.

[62] Vulcani-Freitas T.M., Saba-Silva N., Cappellano A., Cavalheiro S., Marie S.K., Oba-Shinjo S.M., Malheiros S.M., de Toledo S.R. (2011). ASPM gene expression in medulloblastoma. Childs Nerv. Syst..

[63] Feng Z., Zhang J., Zheng Y., Liu J., Duan T., Tian T. (2021). Overexpression of abnormal spindle-like microcephaly-associated (ASPM) increases tumor aggressiveness and predicts poor outcome in patients with lung adenocarcinoma. Transl. Cancer Res..

[64] Kato T.A., Okayasu R., Jeggo P.A., Fujimori A. (2011). ASPM influences DNA double-strand break repair and represents a potential target for radiotherapy. Int. J. Radiat. Biol..

[65] Xu S., Wu X., Wang P., Cao S.L., Peng B., Xu X. (2021). ASPM promotes homologous recombination-mediated DNA repair by safeguarding BRCA1 stability. iScience.

[66] Yu J., Zhong T., Wang J., Xie S., Liu L., Wang M., Wu F., Xiao C., Chen X., Yan W. (2022). ASPM induces radiotherapy resistance by disrupting microtubule stability leading to chromosome malsegregation in non-small cell lung cancer.

