# Supplementary material for: Deciphering the Role of ASPM in Breast Cancer: A Comprehensive Multicohort Study
Source: Cancers (Basel). 2024 Nov 13;16(22):3814. doi: 10.3390/cancers16223814 (PMC11592464; doi:10.3390/cancers16223814)
Supplement: Supplementary file 1 [file cancers-16-03814-s001.zip › cancers-3247414-supplementary.pdf]

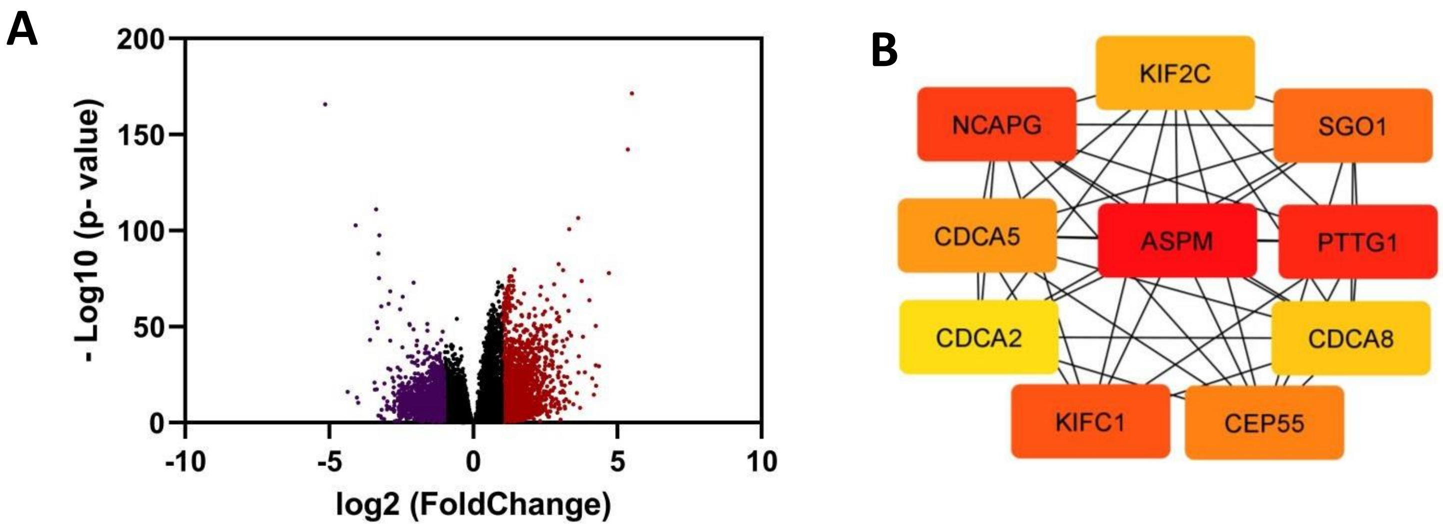

Supplementary Figure S1: (A) Volcano plot of DEGs in TCGA-BC cohort. **(B)** Identification of the hub genes from the PPI network using the density of maximum neighborhood component (DMNC) algorithm. Edges represent the protein-protein associations. The red nodes represent genes with a high sore, while the yellow node represent genes with a low sore. *ASPM* was identified as the gen with the highest score.

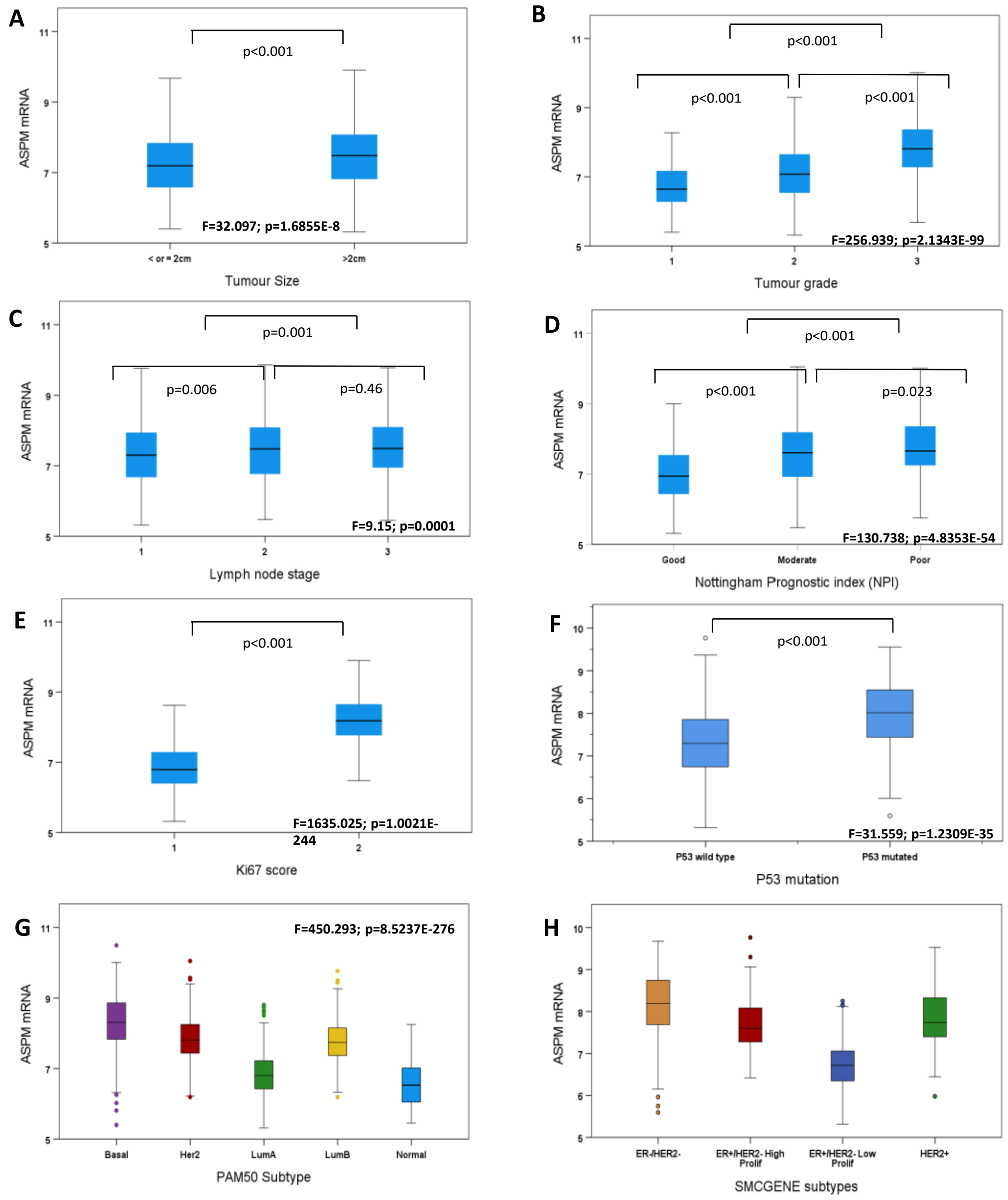

Supplementary Figure S2: *ASPM* mRNA expression and its association with clinicopathological parameters and molecular subtypes: A) *ASPM* and tumor size, B) *ASPM* and tumor grade, C) *ASPM* and lymph node stage, D) *ASPM* and NPI, E) *ASPM* and Ki67 score F) *ASPM* and P53 mutation G) *ASPM* and PAM50 subtypes, H) *ASPM* and SMCGENE subtypes in the METABRIC cohort using One-way ANOVA with post-hoc Tukey test.

Supplementary Figure S3

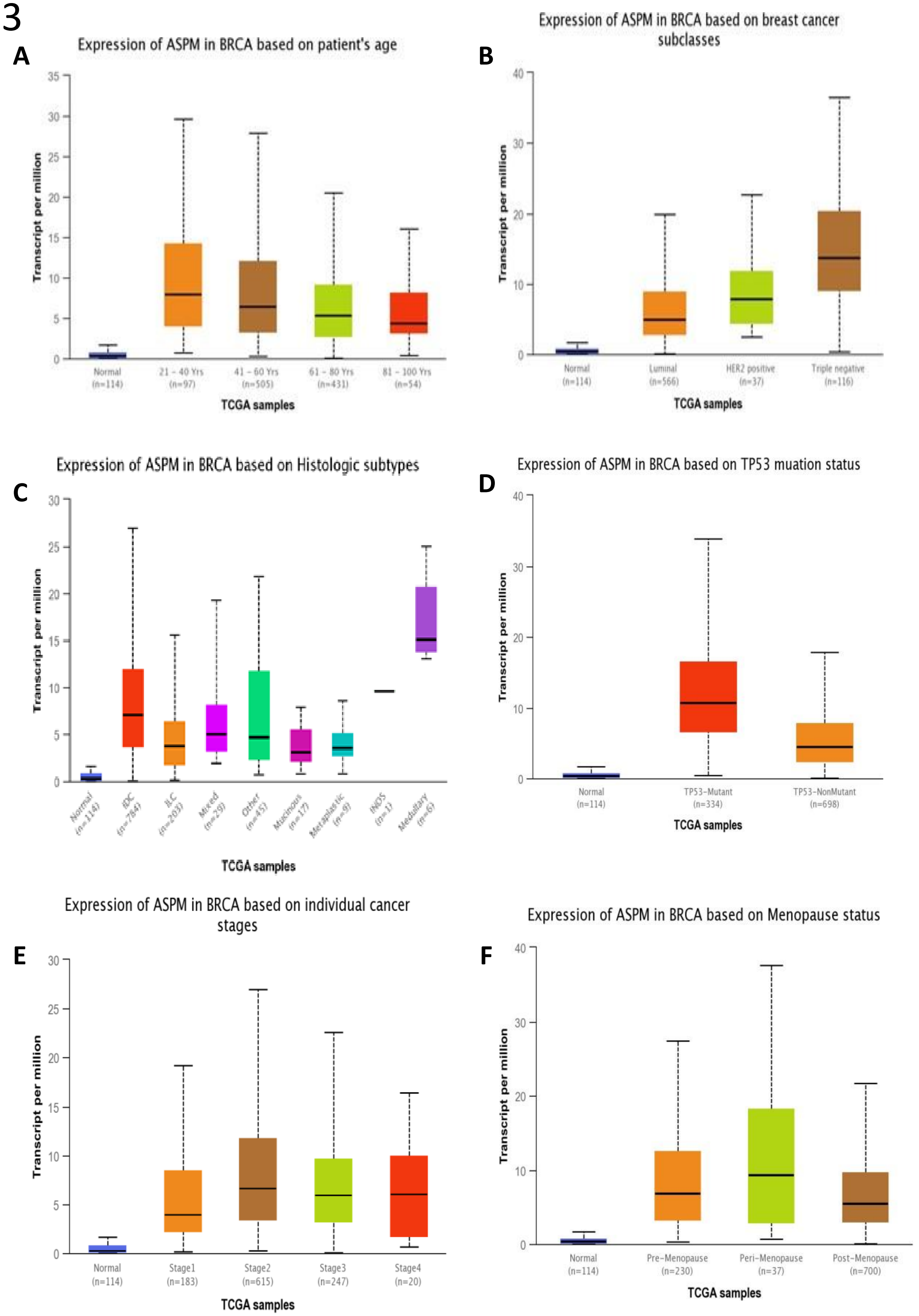

Supplementary Figure S3. *ASPM* mRNA expression and its association with clinicopathological parameters andmolecular subtypes, A) *ASPM* and patient's age, B) *ASPM* and PAM50 BC subtypes, C) *ASPM* and Histological subtypes, D) *ASPM* and TP53 mutation status, E) *ASPM* and lymph node stage, F) *ASPM* and Menopausal status in the TCGA cohort.

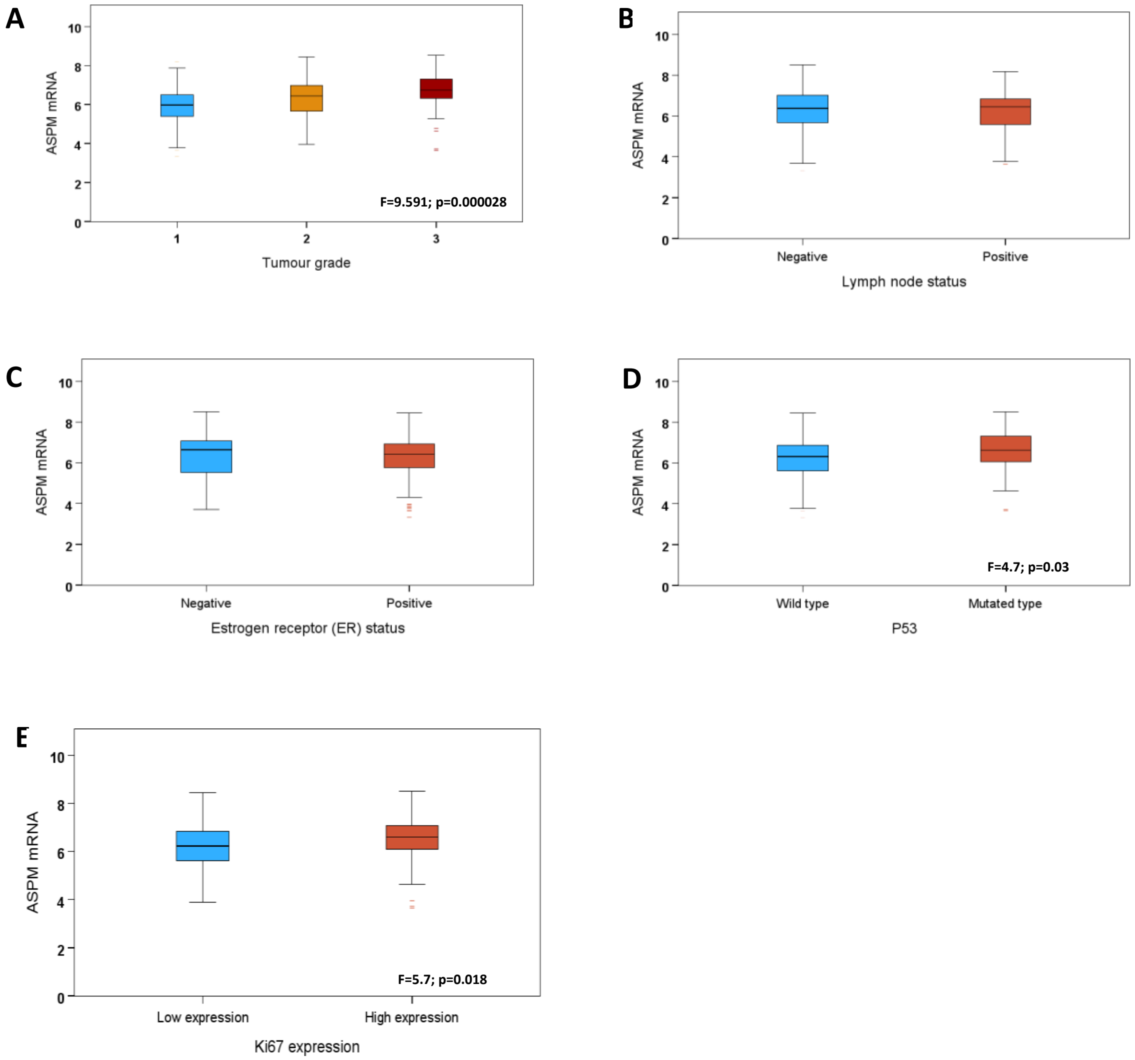

Supplementary Figure S4. *ASPM* mRNA expression and its association with clinicopathological parameters including, A) *ASPM* and tumour grade, B) *ASPM* and lymph node status, C) *ASPM* and Estrogen receptor status, D) *ASPM* and TP53 mutation status, E) *ASPM* and Ki67 status, in the Uppsala cohort.

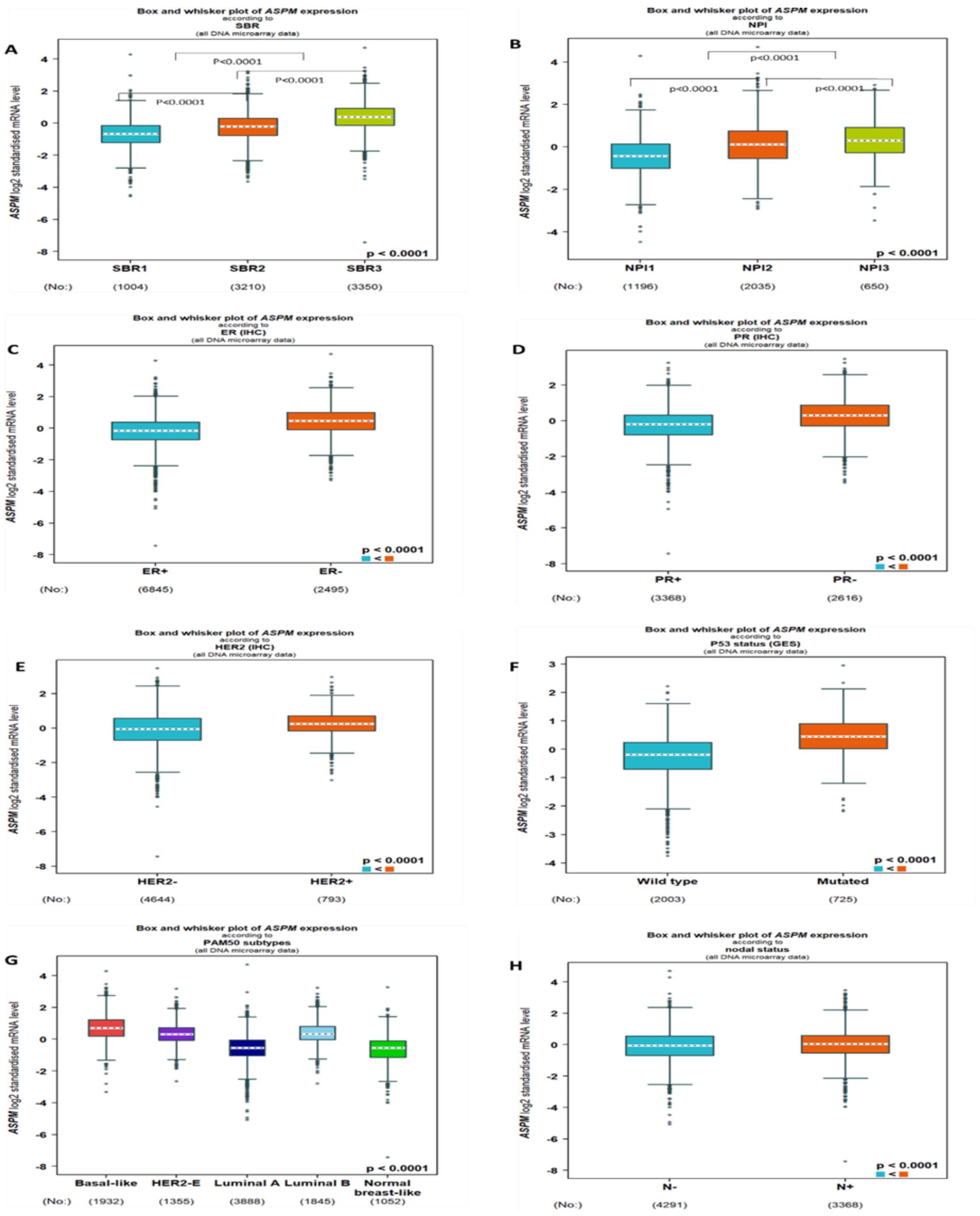

Supplementary Figure S5: *ASPM* expression and its association, with: A) tumor grade, B) NPI, C) ER status, D) PR status, E) HER2 status, F) P53 status, G) PAM50 subtypes, H) nodal status, in the combined multi-centric cohort.

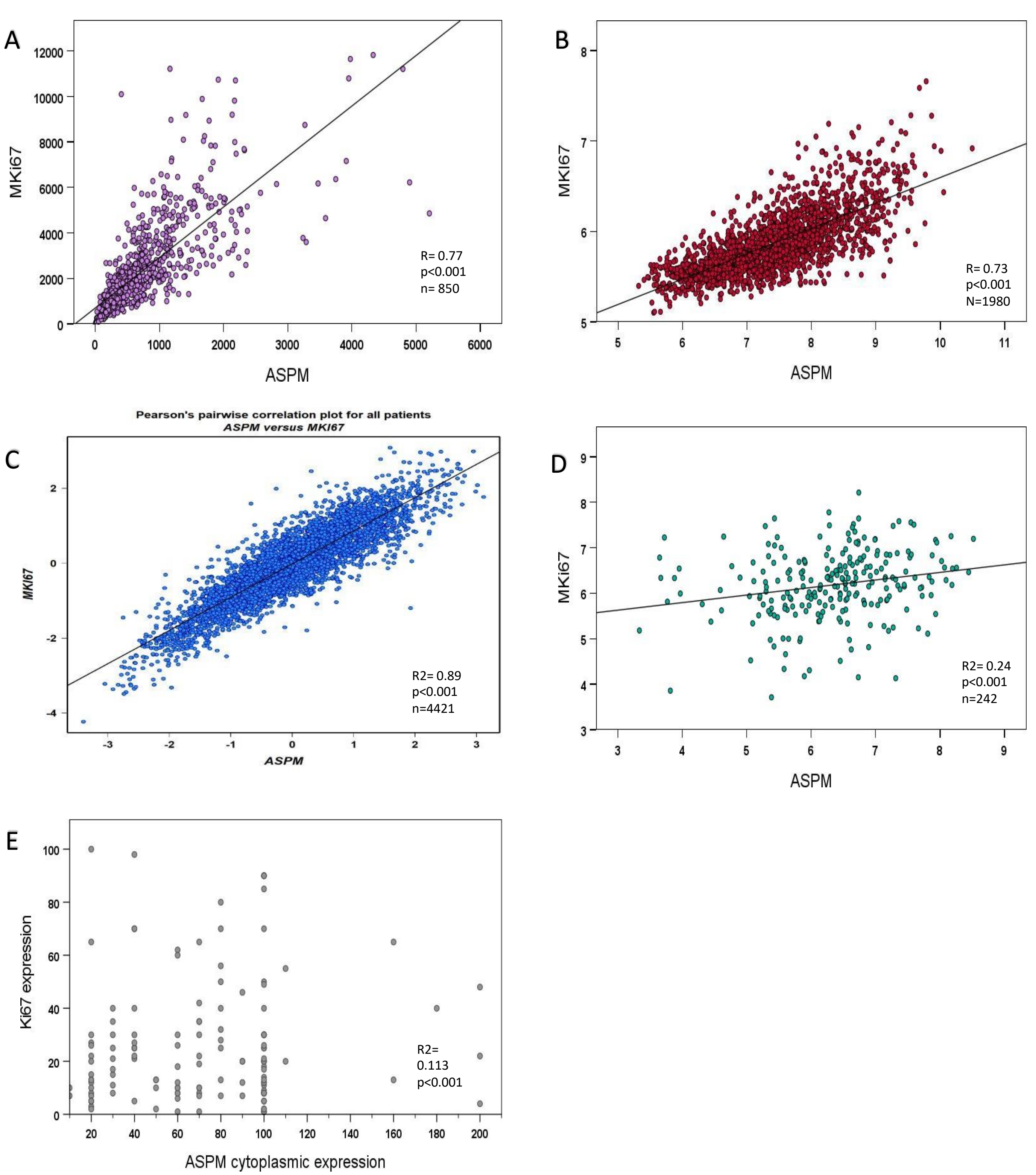

Supplementary Figure S6: The correlation between the *ASPM* mRNA expression and *Ki67* mRNA expression in (A) theTCGA cohort, (B) the METABRIC cohort, (C) the multicentric cohort, (D) the Uppsala cohort, in addition to (E) the correlation between the ASPM cytoplasmic expression and Ki67 protein expression in Nottingham cohort.

Supplementary Tables

Supplementary Table S1. list of all the datasets used to construct the multicentre combined cohort to investigate the expression of *ASPM* transcript (n=7252) in early-stage breast cancer patients using bc-GenExMiner.

Study code	Reference	*ASPM* transcript		
		Number of patients	Number of patients	Number of events disease-free Survival (DFS)
Rosetta2002	Van de Vijver et al., 2002	295	295	122
PNAS1732912100	Sotiriou et al., 2003	99	99	0
GSE1379	Ma et al., 2004	59	59	27
GSE2603	Minn et al., 2005	82	82	27
GSE1456	Pawitan et al., 2005	159	159	50
GSE2034	Wang et al., 2005	286	286	107
GSE2741	Weigelt et al., 2005	50	50	13
GSE3143	Bild et al., 2006	158	158	0
E_TABM_158	Chin et al., 2006	112	112	42
GSE4922	Ivshina et al., 2006	249	249	89
GSE7390	Desmedt et al., 2007	198	198	91
GSE6532	Loi et al., 2007	267	259	88
GSE5327	Minn et al., 2007	58	58	11
E_UCON_1	Naderi et al., 2007	135	135	65
GSE7849	Anders et al., 2008	75	75	0
GSE9893	Chanrion et al., 2008	151	151	55
GSE9195	Loi et al., 2008	77	77	13
GSE10510	Calabrò et al., 2009	139	134	96
GSE11264	Jézéquel et al., 2009	252	252	0
GSE11121	Schmidt et al., 2008	200	200	46
GSE12093	Zhang et al., 2009	136	136	20
GSE8757	Chin et al., 2007	171	171	56
GSE7378	Zhou et al., 2007	54	54	9
GSE16391	Desmedt et al., 2009	55	55	55
GSE22133	Jönsson et al., 2010	346	339	151
GSE19615	Li et al., 2010	115	115	14
GSE17907	Sircoulomb et al., 2010	55	39	17
GSE22219	Buffa et al., 2011	216	216	82
GSE20711	Dedeurwaerder et al., 2011	85	85	36
GSE26971	Filipits et al., 2011	277	258	58
GSE25055	Hatzis et al., 2011	309	309	65

GSE20685	Kao et al., 2011	296	296	73
GSE21653	Sabatier et al., 2011	239	229	74
GSE16987	Wang et al., 2011	149	147	10
GSE45255	Nagalla et al., 2013	41	41	14
GSE2109	expO et al., 2005	298	0	0
GSE8193	Yau et al., 2007	47	0	0
GSE20462	Parris et al., 2010	94	94	44
GSE17705	Symmans et al., 2010	43	43	8
GSE24450	Heikkinen et al., 2011	174	174	34
GSE31448	Sabatier et al., 2011	71	0	0
E_MTAB_365	Guedj et al., 2012	536	526	118
GSE30682	Servant et al., 2012	343	0	0
GSE42568	Clarke et al., 2013	104	104	48
GSE40115	Larsen et al., 2013	183	0	0
GSE55348	Castagnoli et al., 2014	53	53	23
GSE43358	Fumagalli et al., 2014	56	55	10
GSE36295	Merdad et al., 2014	45	0	0
GSE37751	Terunuma et al., 2014	55	55	19
GSE76274	Burstein et al., 2015	66	0	0
GSE97177	Biermann et al., 2017	53	0	0
GSE12276	Bos et al., 2009	204	204	204
GSE18864	Silver et al., 2010	75	0	0
GSE80999	Aure et al., 2017	381	0	0
GSE86166	Prabhakaran et al., 2017	366	366	119
		8892	7252	2303

Supplementary Table S2: list of all the datasets used to construct the multicenter combined cohort with different microarrays platforms used (using bc-GenExMiner)

Study code	Reference		Number of patients	Microarray platforms	DNA chip	Number of unique genes (2022)	Processing *
	Author	Year					
**Rosetta2002**	Van de Vijver *et al.,*	2002	295	Agilent	25k oligo custom	14 799	log2 ratio
**PNAS1732912100**	Sotiriou *et al.,*	2003	99	NCI	8k cDNA custom	4 336	log2 ratio
**GSE1379**	Ma *et al.,*	2004	59	Arcturus	22k oligo custom	14 800	log2 ratio
**GSE2603**	Minn *et al.,*	2005	82	Affymetrix™	HG-U133A	12 629	MAS5 and log2
**GSE1456**	Pawitan *et al.,*	2005	159	Affymetrix™	HG-U133A + B	18 163	MAS5 and log2
**GSE2034**	Wang *et al.,*	2005	286	Affymetrix™	HG-U133A	12 629	MAS5 and log2
**GSE2741**	Weigelt *et al.,*	2005	50	Agilent	Human 1A oligo UNC custom	13 955	log2 ratio
**GSE3143**	Bild *et al.,*	2006	158	Affymetrix™	HG-U95A v2	8 749	MAS5 and log2
**E_TABM_158**	Chin *et al.,*	2006	112	Affymetrix™	HG-U133A v2	12 629	MAS5 and log2
**GSE4922**	Ivshina *et al.,*	2006	249	Affymetrix™	HG-U133A + B	18 163	MAS5 and log2
**GSE7390**	Desmedt *et al.,*	2007	198	Affymetrix™	HG-U133A	12 629	MAS5 and log2
**GSE6532**	Loi *et al.,*	2007	267	Affymetrix™	HG U133A + B + P2	20 126	MAS5 and log2
**GSE5327**	Minn *et al.,*	2007	58	Affymetrix™	HG-U133A	12 629	MAS5 and log2
**E_UCON_1**	Naderi *et al.,*	2007	135	Agilent	Human 1A oligo G4110A	14 233	log2 ratio
**GSE7849**	Anders *et al.,*	2008	75	Affymetrix™	HG-U95A v2	8 749	MAS5 and log2
**GSE9893**	Chanrion *et al.,*	2008	151	MLRG	Human 21k v12.0	14 959	log2 ratio
**GSE9195**	Loi *et al.,*	2008	77	Affymetrix™	HG-U133P2	20 126	MAS5 and log2
**GSE10510**	Calabrò *et al.,*	2009	139	DKFZ	35k oligo	17 770	log2 ratio
**GSE11264**	Jézéquel *et al.,*	2009	252	UMGC-IRCNA	9k cDNA custom	1 807	log2 ratio
**GSE11121**	Schmidt *et al.,*	2008	200	Affymetrix™	HG-U133A	12 629	MAS5 and log2
**GSE12093**	Zhang *et al.,*	2009	136	Affymetrix™	HG-U133A	12 629	MAS5 and log2
**GSE8757**	Chin *et al.,*	2007	171	VUMC Microarray	Human 30K 60-mer oligo array	17 688	log2 ratio
**GSE7378**	Zhou *et al.,*	2007	54	Affymetrix™	HG-U133A	12 629	MAS5 and log2
**GSE16391**	Desmedt *et al.,*	2009	55	Affymetrix™	HG-U133P2	20 126	MAS5 and log2
**GSE22133**	Jönsson *et al.,*	2010	346	SweGene	H_v2.1.1 55K	9 222	log2 ratio
**GSE19615**	Li *et al.,*	2010	115	Affymetrix™	HG-U133P2	20 126	MAS5 and log2
**GSE17907**	Sircoulomb *et al.,*	2010	55	Affymetr	HG-U133P2	20 126	MAS5 and

				ix™			log2
**GSE22219**	Buffa *et al.,*	2011	216	Illumina	HumanRef-8 v1.0 expr-bc	15 729	log2 ratio
**GSE20711**	Dedeurwaerder *et al.,*	2011	85	Affymetrix™	HG-U133P2	20 126	MAS5 and log2
**GSE26971**	Filipits *et al.,*	2011	277	Affymetrix™	HG-U133A	12 629	MAS5 and log2
**GSE25055**	Hatzis *et al.,*	2011	309	Affymetrix™	HG-U133A	12 629	MAS5 and log2
**GSE20685**	Kao *et al.,*	2011	296	Affymetrix™	HG-U133P2	20 126	MAS5 and log2
**GSE21653**	Sabatier *et al.,*	2011	239	Affymetrix™	HG-U133P2	20 126	MAS5 and log2
**GSE16987**	Wang *et al.,*	2011	149	Illumina	HumanRef-8 v2.0 expr-bc	16 741	log2 ratio
**GSE33926**	Kuo *et al.,*	2012	51	Agilent	Human 1A Microarray (V2) G4110B	16 608	log2 ratio
**GSE45255**	Nagalla *et al.,*	2013	41	Affymetrix™	HG-U133A	12 629	MAS5 and log2
**GSE2109**	expO *et al.,*	2005	298	Affymetrix™	HG-U133P2	20 126	MAS5 and log2
**GSE8193**	Yau *et al.,*	2007	47	Affymetrix™	HG-U133A	12 629	MAS5 and log2
**GSE20462**	Parris *et al.,*	2010	94	Illumina	HumanHT-12 V3.0	18 948	Quantile norm. and log2
**GSE17705**	Symmans *et al.,*	2010	43	Affymetrix™	HG-U133A	12 629	MAS5 and log2
**GSE24450**	Heikkinen *et al.,*	2011	174	Illumina	HumanHT-12 V3.0	18 948	Quantile norm. and log2
**GSE31448**	Sabatier *et al.,*	2011	71	Affymetrix™	HG-U133P2	20 126	MAS5 and log2
**METABRIC**	Curtis *et al.,*	2012	1 980	Illumina	HumanHT-12 V3.0	17 962	Quantile norm. and log2
**E_MTAB_365**	Guedj *et al.,*	2012	536	Affymetrix™	HG-U133P2	20 126	MAS5 and log2
**GSE30682**	Servant *et al.,*	2012	343	Illumina	HumanWG-6 v3.0	18 948	Quantile norm. and log2
**GSE42568**	Clarke *et al.,*	2013	104	Affymetrix™	HG-U133P2	20 126	MAS5 and log2
**GSE40115**	Larsen *et al.,*	2013	183	Agilent	SurePrint G3 Human GE 8x60K	19 966	log2 ratio
**GSE55348**	Castagnoli *et al.,*	2014	53	Illumina	HumanHT-12 WG-DASL V4.0 R2	18 894	Quantile norm. and log2
**GSE43358**	Fumagalli *et al.,*	2014	56	Affymetrix™	HG-U133P2	20 126	MAS5 and log2
**GSE36295**	Merdad *et al.,*	2014	45	Affymetrix™	Gene 1.0 ST	19 944	rma-gene-level
**GSE37751**	Terunuma *et al.,*	2014	55	Affymetrix™	Gene 1.0 ST	19 944	rma-gene-level
**GSE76274**	Burstein *et al.,*	2015	66	Affymetrix™	HG-U133P2	20 126	MAS5 and log2
**GSE97177**	Biermann *et al.,*	2017	53	Illumina	HumanHT-12 V3.0	18 948	Quantile norm. and log2
**GSE12276**	Bos *et al.,*	2009	204	Affymetrix™	HG-U133P2	20 126	MAS5 and log2
**GSE12276**	Bos *et al.,*	2009	204	Affymetrix™	HG-U133P2	20 126	MAS5 and log2

**GSE18864**	Silver *et al.,*	2010	75	Affymetrix™	HG-U133P2	20 126	MAS5 and log2
**GSE18864**	Silver *et al.,*	2010	75	Affymetrix™	HG-U133P2	20 126	MAS5 and log2
**GSE76124**	Burstein *et al.,*	2015	198	Affymetrix™	HG-U133P2	20 126	MAS5 and log2
**GSE58812**	Jézéquel *et al.,*	2015	107	Affymetrix™	HG-U133P2	20 126	MAS5 and log2
**GSE83937**	Jézéquel *et al.,*	2019	131	Affymetrix™	HG-U133P2	20 126	MAS5 and log2
**GSE80999**	Aure *et al.,*	2017	381	Agilent	SurePrint G3 Human GE 8x60K	18 595	log2 ratio
**GSE86166**	Prabhakaran *et al.,*	2017	366	Affymetrix™	Rosetta/Merck human RSTA custom Affymetrix 2.0 microarray	19 476	rma-gene-level
**GSE95700**	Tseng *et al.,*	2017	56	Affymetrix™	HG-U133P2	20 126	MAS5 and log2
**GSE114168**	Romero *et al.,*	2018	53	Affymetrix™	HG-U133P2	20 126	MAS5 and log2
**GSE135565**	Kim *et al.,*	2020	84	Affymetrix™	HG-U133P2	20 126	MAS5 and log2

*Data have been converted to a common scale (median equal to 0 and standard deviation equal to 1).

Supplementary Table S3: Preprocessing steps for multicenter combined cohort with different data types using bc-GenExMiner.

Data Type	Pre-processing Step	Description
DNA Microarrays	Affymetrix	Raw CEL data were MAS5.0-normalized using Affymetrix Expression Console™, except for Gene 1.0 ST which was pre-processed using a robust multiarray analysis (RMA) algorithm from the Affy Bioconductor package. (a)
	Non-Affymetrix	Data were downloaded and processed, including patient-to-reference ratio and log2-transformation if not calculated.
	Merging	Data from all studies, except TNBC subtypes, were converted to a common scale (median = 0, standard deviation = 1). TNBC cohorts were standardized using the ComBat method.
RNA-seq Data	TCGA	RNA-Seq dataset downloaded from TCGA. Alignment using STAR two-pass method counts normalized using FPKM. FPKM values log2-transformed with an offset of 0.1.
	TCGA (Nature)	Processed RNA-seq data from TCGA. TPM values were obtained from GEO accession numbers GSM1536837 (tumour) and GSM1697009 (tumour-adjacent). FPKM values normalized to TPM using edgeR and limma. Log2-transformed with an offset of 1.
	GTEx	Gene expression values for healthy tissues from the GTEx project. FPKM values normalized using the Rsubread package and hg19 as a reference genome. Converted to TPM with an offset of 1 before log2 transformation.
	SCAN-B (GSE81540)	RNA-seq reads mapped to hg19 with tophat2, normalized in FPKM using cufflinks2 pipeline, log2-transformed with an offset of 0.1.
	Merging	Studies data were converted to a common scale (median = 0, standard deviation = 1). For analysis of tissue nature, no standardization is required due to consistent processing using the Rsubread package and alignment to the same reference genome. TNBC standardized using ComBat.

Supplementary Table S4: The REMARK checklist

Items to be reported		Page no.
INTRODUCTION		
1	State the marker examined, the study objectives, and any pre-specified hypotheses.	3,4
MATERIALS AND METHODS		
Patients		
2	Describe the characteristics (e.g., disease stage or co-morbidities) of the study patients, including their source and inclusion and exclusion criteria.	4
3	Describe treatments received and how chosen (e.g., randomized or rule-based).	4,5
Specimen characteristics		
4	Describe the type of biological material used (including control samples) and methods of preservation and storage.	5,6
Assay methods		
5	Specify the assay method used and provide (or reference) a detailed protocol, including specific reagents or kits used, quality control procedures, reproducibility assessments, quantitation methods, and scoring and reporting protocols. Specify whether and how assays were performed blinded to the study endpoint.	5,6
Study design		
6	State the method of case selection, including whether prospective or retrospective and whether stratification or matching (e.g., by stage of disease or age) was used. Specify the time from which cases were taken, the end of the follow-up period, and the median follow-up time.	4
7	Precisely define all clinical endpoints examined.	4
8	List all candidate variables initially examined or considered for inclusion in models.	
9	Give rationale for sample size; if the study was designed to detect a specified effect size, give the target power and effect size.	
Statistical analysis methods		
10	Specify all statistical methods, including details of any variable selection procedures and other model-building issues, how model assumptions were verified, and how missing data were handled.	6,7
11	Clarify how marker values were handled in the analyses; if relevant, describe methods used for cut-point determination.	6,7
RESULTS		
Data		

12	Describe the flow of patients through the study, including the number of patients included in each stage of the analysis (a diagram may be helpful) and reasons for dropout. Specifically, both overall and for each subgroup extensively examined report the numbers of patients and the number of events.	7,8
13	Report distributions of basic demographic characteristics (at least age and sex), standard (disease-specific) prognostic variables, and tumour markers, including numbers of missing values.	7,8
Analysis and presentation		
14	Show the relation of the marker to standard prognostic variables.	7
15	Present univariable analyses showing the relation between the marker and outcome, with the estimated effect (e.g., hazard ratio and survival probability). Preferably provide similar analyses for all other variables being analysed. For the effect of a tumour marker on a time-to-event outcome, a Kaplan-Meier plot is recommended.	8,9
16	For key multivariable analyses, report estimated effects (e.g., hazard ratio) with confidence intervals for the marker and, at least for the final model, all other variables in the model.	9
17	Among reported results, provide estimated effects with confidence intervals from an analysis in which the marker and standard prognostic variables are included, regardless of their statistical significance.	9
18	If done, report the results of further investigations, such as checking assumptions, sensitivity analyses, and internal validation.	
DISCUSSION		
19	Interpret the results in the context of the pre-specified hypotheses and other relevant studies; include a discussion of the limitations of the study.	10-13
20	Discuss implications for future research and clinical value.	10-13

Supplementary Table S5: Genes enriched in the cell division using Gene Ontology (GO) analysis of DAVID online tool.

converted_alias	name	description
ENSG00000066279	ASPM	assembly factor for spindle microtubules [Source:HGNC Symbol;Acc:HGNC:19048]
ENSG00000169679	BUB1	BUB1 mitotic checkpoint serine/threonine kinase [Source:HGNC Symbol;Acc:HGNC:1148]
ENSG00000080986	NDC80	NDC80 kinetochore complex component [Source:HGNC Symbol;Acc:HGNC:16909]
ENSG00000143228	NUF2	NUF2 component of NDC80 kinetochore complex [Source:HGNC Symbol;Acc:HGNC:14621]
ENSG00000129195	PIMREG	PICALM interacting mitotic regulator [Source:HGNC Symbol;Acc:HGNC:25483]
ENSG00000073921	PICALM	phosphatidylinositol binding clathrin assembly protein [Source:HGNC Symbol;Acc:HGNC:15514]
ENSG00000164611	PTTG1	PTTG1 regulator of sister chromatid separation, securin [Source:HGNC Symbol;Acc:HGNC:9690]
ENSG00000088325	TPX2	TPX2 microtubule nucleation factor [Source:HGNC Symbol;Acc:HGNC:1249]
ENSG00000011426	ANLN	anillin, actin binding protein [Source:HGNC Symbol;Acc:HGNC:14082]
ENSG00000087586	AURKA	aurora kinase A [Source:HGNC Symbol;Acc:HGNC:11393]
ENSG00000178999	AURKB	aurora kinase B [Source:HGNC Symbol;Acc:HGNC:11390]
ENSG00000089685	BIRC5	baculoviral IAP repeat containing 5 [Source:HGNC Symbol;Acc:HGNC:593]
ENSG00000117399	CDC20	cell division cycle 20 [Source:HGNC Symbol;Acc:HGNC:1723]
ENSG00000164045	CDC25A	cell division cycle 25A [Source:HGNC Symbol;Acc:HGNC:1725]
ENSG00000094804	CDC6	cell division cycle 6 [Source:HGNC Symbol;Acc:HGNC:1744]
ENSG00000184661	CDCA2	cell division cycle associated 2 [Source:HGNC Symbol;Acc:HGNC:14623]
ENSG00000111665	CDCA3	cell division cycle associated 3 [Source:HGNC Symbol;Acc:HGNC:14624]
ENSG00000146670	CDCA5	cell division cycle associated 5 [Source:HGNC Symbol;Acc:HGNC:14626]
ENSG00000134690	CDCA8	cell division cycle associated 8 [Source:HGNC Symbol;Acc:HGNC:14629]
ENSG00000115163	CENPA	centromere protein A [Source:HGNC Symbol;Acc:HGNC:1851]
ENSG00000203760	CENPW	centromere protein W [Source:HGNC Symbol;Acc:HGNC:21488]
ENSG00000138180	CEP55	centrosomal protein 55 [Source:HGNC Symbol;Acc:HGNC:1161]
ENSG00000167513	CDT1	chromatin licensing and DNA replication factor 1 [Source:HGNC Symbol;Acc:HGNC:24576]
ENSG00000145386	CCNA2	cyclin A2 [Source:HGNC Symbol;Acc:HGNC:1578]
ENSG00000157456	CCNB2	cyclin B2 [Source:HGNC Symbol;Acc:HGNC:1580]
ENSG00000170312	CDK1	cyclin dependent kinase 1 [Source:HGNC Symbol;Acc:HGNC:1722]
ENSG00000101447	FAM83D	family with sequence similarity 83 member D [Source:HGNC

		Symbol;Acc:HGNC:16122]
ENSG00000105255	FSD1	fibronectin type III and SPRY domain containing 1 [Source:HGNC Symbol;Acc:HGNC:13745]
ENSG00000186185	KIF18B	kinesin family member 18B [Source:HGNC Symbol;Acc:HGNC:27102]
ENSG00000142945	KIF2C	kinesin family member 2C [Source:HGNC Symbol;Acc:HGNC:6393]
ENSG00000237649	KIFC1	kinesin family member C1 [Source:HGNC Symbol;Acc:HGNC:6389]
ENSG00000109805	NCAPG	non-SMC condensin I complex subunit G [Source:HGNC Symbol;Acc:HGNC:24304]
ENSG00000121152	NCAPH	non-SMC condensin I complex subunit H [Source:HGNC Symbol;Acc:HGNC:1112]
ENSG00000166851	PLK1	polo like kinase 1 [Source:HGNC Symbol;Acc:HGNC:9077]
ENSG00000129810	SGO1	shugoshin 1 [Source:HGNC Symbol;Acc:HGNC:25088]
ENSG00000154839	SKA1	spindle and kinetochore associated complex subunit 1 [Source:HGNC Symbol;Acc:HGNC:28109]
ENSG00000165480	SKA3	spindle and kinetochore associated complex subunit 3 [Source:HGNC Symbol;Acc:HGNC:20262]
ENSG00000175063	UBE2C	ubiquitin conjugating enzyme E2 C [Source:HGNC Symbol;Acc:HGNC:15937]
